# Characteristics of Extracellular Vesicles Released by the Pathogenic Yeast-Like Fungi *Candida glabrata*, *Candida parapsilosis* and *Candida tropicalis*

**DOI:** 10.3390/cells9071722

**Published:** 2020-07-18

**Authors:** Justyna Karkowska-Kuleta, Kamila Kulig, Elzbieta Karnas, Ewa Zuba-Surma, Olga Woznicka, Elzbieta Pyza, Patryk Kuleta, Artur Osyczka, Maria Rapala-Kozik, Andrzej Kozik

**Affiliations:** 1 Department of Comparative Biochemistry and Bioanalytics, Faculty of Biochemistry, Biophysics and Biotechnology, Jagiellonian University, Gronostajowa 7, 30-387 Krakow, Poland; maria.rapala-kozik@uj.edu.pl; 2 Department of Analytical Biochemistry, Faculty of Biochemistry, Biophysics and Biotechnology, Jagiellonian University, Gronostajowa 7, 30-387, Kraków, Poland; kamila.kulig@uj.edu.pl (K.K.); andrzej.kozik@uj.edu.pl (A.K.); 3 Department of Cell Biology, Faculty of Biochemistry, Biophysics and Biotechnology, Jagiellonian University, Gronostajowa 7, 30-387 Kraków, Poland; e.karnas@uj.edu.pl (E.K.); ewa.zuba-surma@uj.edu.pl (E.Z.-S.); 4 Laboratory of Stem Cell Biotechnology, Malopolska Centre of Biotechnology, Jagiellonian University, Gronostajowa 7a, 30-387 Krakow, Poland; 5 Department of Cell Biology and Imaging, Institute of Zoology and Biomedical Research, Jagiellonian University, Gronostajowa 9, 30-387 Krakow, Poland; olga.woznicka@uj.edu.pl (O.W.); elzbieta.pyza@uj.edu.pl (E.P.); 6 Department of Molecular Biophysics, Faculty of Biochemistry, Biophysics and Biotechnology, Jagiellonian University, Gronostajowa 7, 30-387 Kraków, Poland; patryk.kuleta@uj.edu.pl (P.K.); artur.osyczka@uj.edu.pl (A.O.)

**Keywords:** extracellular vesicles, pathogenic fungi, candidiasis, moonlighting proteins, non-classical secretion

## Abstract

*Candida* spp. yeast-like fungi are opportunistic pathogens in humans and have been recently found to release extracellular vesicles (EVs) that are involved in many vital biological processes in fungal cells. These include communication between microorganisms and host–pathogen interactions during infection. The production of EVs and their content have been significantly characterized in the most common candidal species *Candida albicans*, including the identification of numerous virulence factors and cytoplasmic proteins in the EV cargo. We have here conducted the isolation and proteomic characterization of EVs produced by the clinically important non-albicans *Candida* species *C. glabrata*, *C. tropicalis* and *C. parapsilosis*. With the use of ultracentrifugation of the cell-free culture supernatant, the candidal EVs were collected and found to be a heterogeneous population of particles for each species with sizes ranging from 60–280 nm. The proteinaceous contents of these vesicles were analyzed using LC-MS/MS, with particular attention paid to surface-expressed proteins that would come into immediate and direct contact with host cells. We thereby identified 42 extracellular and surface-connected proteins from *C. glabrata*, 33 from *C. parapsilosis*, and 34 from *C. tropicalis,* including membrane-associated transporters, glycoproteins and enzymes involved in the organization of the fungal cell wall, as well as several cytoplasmic proteins, including alcohol dehydrogenase, enolase, glyceraldehyde-3-phosphate dehydrogenase, phosphoglycerate kinase and pyruvate kinase, for which the vesicular transport is a possible mechanism underlying their non-classical secretion.

## 1. Introduction

Polymorphous fungi of the *Candida* genus are still considered to be the main fungal infectious agents in humans, causing invasive mycoses and bloodstream infections that threaten the health and life of diverse groups of vulnerable patients. Groups that are particularly susceptible to poor outcomes from these infections include infants, the elderly, and individuals with impaired immunity or weakened defense mechanisms due to systemic diseases such as diabetes or cancer, or who have had medical procedures such as surgery or the application of parenteral nutrition or central venous catheters [1,2]. The wide distribution of these opportunistic pathogens in the environment, their relatively easy transmission between individuals, difficulties with making an accurate and rapid diagnosis, and the ineffective treatment of invasive candidiases related to acquired and naturally emerging pathogen resistance to antifungal drugs, result in high morbidity rates and significant mortality outcomes of up to 30–40% [3,4,5,6,7]. The burden of severe fungal infections worldwide and the related threats to the constantly increasing number of individuals susceptible to such infections, requires a comprehensive investigation of their pathogenesis. Although the best-known and the most common species of this genus causing infections in humans is still *C. albicans*, the global epidemiology of candidiasis is currently changing and other species, collectively called non-*albicans Candida* (NAC) species and primarily including *C. glabrata*, *C. parapsilosis* and *C. tropicalis*, are increasingly emerging as etiological agents of systemic diseases [8,9,10]. The biology and the virulence mechanisms of NAC species are still not thoroughly understood, which is a particularly important and pressing issue if one takes into account the growing clinical significance of these fungi and the increasing number of serious infections they cause [1].

One of the recently described mechanisms considered as essential not only in fungal cell physiology and intraspecies communication, but also in the virulence of fungal pathogens, is the production of extracellular vesicles (EVs). These are structures surrounded by a lipid bilayer, carrying various proteins, nucleic acids, carbohydrates, toxins, pigments and other molecules in their interior or on their surface, and are involved in the transport of this complex cargo from the inside of the cell to the surrounding environment across the fungal cell wall [11,12,13,14,15,16,17]. Fungal EVs can often be considered as tightly packed compartments, i.e., encapsulated sections of the cytoplasm additionally enriched with selected molecules as a result of external conditions, that deliver their content to the proper destination whilst protecting it from the degradation during transport and ensuring a sufficient concentration of specific co-transported molecules and the possibility of their cooperation at the target site [18,19,20,21]. The production of EVs has been reported for several species of fungal pathogens, including *C. albicans, Cryptococcus neoformans*, *Histoplasma capsulatum, Paracoccidioides brasiliensis, Malassezia sympodialis* and *Alternaria infectoria*, and their contents have been characterized, including the repeat identification of numerous virulence factors and molecules involved in cellular response to stress or adaptation to new environmental conditions [22,23,24,25,26,27,28]. The presence of proteins related to the pathogenesis and development of fungal infection, such as adhesins, immunogens, pigments-synthesizing enzymes, proteases and other hydrolases, in EV preparations undeniably indicates their significant importance in the interactions between invading microorganisms and their human host [29,30]. These relationships are associated with the possibility of the internalization of EVs by phagocytes and with the modulation of the inflammatory response resulting in the production of nitric oxide, IL-4, IL-12, IL-10, TGF-β and TNF-α by immune cells [31,32,33].

Among the *Candida* fungi, most of information available on vesicle production is for *C. albicans* [26,27,30,34]. For other species of this genus with great epidemiological and clinical significance, including *C. glabrata*, *C. tropicalis* and *C. parapsilosis*, there are no data on these structures in spite of their potential biological and pathological importance. Hence, the aim of our present study was the isolation and characterization of EVs produced by *C. glabrata*, *C. tropicalis* and *C. parapsilosis*, with a particular focus on proteomic analysis and the proteins exposed at the surface of EVs that come into immediate and direct contact with host cells.

## 2. Materials and Methods

### 2.1. Fungal Strains and Growth Conditions

*C. glabrata* (Anderson) Meyer et Yarrow strain CBS138 (ATCC^®^2001™), *C. tropicalis* (Castellani) Berkhout strain T1 (ATCC^®^MYA-3404™) and *C. parapsilosis* (Ashford) Langeron et Talice strain CDC 317 (ATCC^®^MYA-4646™) were purchased from American Type Culture Collection (Manassas, VA, USA). Cells were routinely cultured in YPD medium (1% yeast extract, 2% soybean peptone and 2% glucose, Sigma, St. Louis, MO, USA); 5 × 10^7^ cells of each species were first inoculated into 20 mL of YPD broth for growth for 18 h at 30 °C on an orbital rotary shaker MaxQ 6000 (170 rpm) (Thermo Fisher Scientific, Waltham, MA, USA) and then all grown cells were harvested by centrifugation (5000 *g*, 3 min) and 2.5 × 10^10^ (*C. glabrata*), 3.6 × 10^10^ (*C. parapsilosis*) and 1.1 × 10^10^ (*C. tropicalis*) cells were inoculated into 400 mL of RPMI 1640 medium (Biowest, Nuaillé, France) and further cultured for 72 h at 37 °C (170 rpm). Cell numbers were determined by optical density measurements at 600 nm and by colony forming units (CFU) counted after application of aliquots of the culture media on YPD agar plates and further growth for 48 h at 30 °C.

### 2.2. Isolation of Extracellular Vesicles

To isolate EVs, supernatants from aerobic cultures of *C. glabrata*, *C. tropicalis* and *C. parapsilosis* grown in RPMI 1640 medium were collected by centrifugation at 4000 *g* for 15 min at 4 °C, repeated twice, and the pellet containing cells and their remnants was discarded each time. The supernatants were then concentrated 400-fold using an Amicon Ultra-15 Centrifugal Filter Unit with a 100 kDa cut off (Merck, Darmstadt, Germany) with the addition of cOmplete Protease Inhibitor Cocktail (Roche, Basel, Switzerland). The samples were centrifuged for 5 min at 5000 *g* and after discarding the pellet were filtered using an Ultrafree-CL Centrifugal Filter with pore size of 0.65 µm (Merck). After this step, the aliquots were spread onto YPD agar plates to verify the absence of any remaining fungal cells. Concentrated supernatants were then ultracentrifuged at 4 °C for 1 h at a rotor speed of 40,000 rpm, which corresponds to relative centrifugal field of 146,000 *g* (*k* factor 108), using a fixed-angle type 50.2 Ti Rotor and polycarbonate thick wall centrifuge tubes (13 × 64 mm) with a 13 mm diameter Delrin tube adapters in an Optima^TM^ L-90K Ultracentrifuge (all from Beckman Coulter, Brea, CA, USA). The obtained EV pellets were gently washed with 400 µL of 0.22 µm-filtered phosphate buffered saline (PBS) buffer, pH 7.4 and subjected to another ultracentrifugation step in 400 µL of PBS under the same conditions. The supernatant was then discarded and the EVs were transferred in 200 µL of PBS to Eppendorf tubes and frozen at −80 °C until further use.

### 2.3. EV Size and Concentration Measurements

The size and concentration of the EVs prepared from *C. glabrata*, *C. tropicalis* and *C. parapsilosis* were measured using the nanoparticle tracking analysis (NTA) and NanoSight NS300 system with camera type sCMOS, laser Blue488 and NTA software Version 3.4 (Malvern Instruments, Malvern, UK). The samples from two different isolations were recorded three times for 60 s with camera level of 13 and the threshold parameter set on 2. Representative histograms of size distribution were selected for presentation. Measurements were conducted at 25 °C in PBS buffer filtered through a 0.22 µm filter.

### 2.4. Protein and Phospholipid Concentration Measurements

Protein concentrations in the EV-containing samples was measured in five biological replicates for each species with *o*-phthalaldehyde (OPA; Sigma) according to previously described methods [35,36]. In brief, 10 µL of sample and 300 µL of OPA reagent were applied to the wells of a black 96-well microplate (Greiner Bio-One, Monroe, NC, USA) and the fluorescence intensity was measured with excitation and emission wavelengths of 340 and 455 nm, respectively, using a Synergy H1 Microplate Reader (BioTek Instruments, Winooski, VT, USA). The phospholipid concentrations in the EV preparations were measured in six biological replicates for each species using the Phospholipid Assay Kit (Sigma) strictly in accordance with the manufacturer’s instructions.

### 2.5. Visualization of EVs

To visualize the EVs obtained from *C. glabrata*, *C. tropicalis* and *C. parapsilosis*, negative stained transmission electron microscopy (TEM) was used with formvar coated, 300 mesh copper grids prepared for each EV preparation using 2% uranyl acetate (Chemapol, Prague, Czech Republic). The JEOL JEM2100 HT transmission electron microscope was then used to observe the samples (JEOL, Tokyo, Japan).

### 2.6. Liquid Chromatography-Coupled Tandem Mass Spectrometry (LC-MS/MS) Identification of Proteins Localized Within and at the Surface of the EVs

To identify proteins localized at the surface of fungal EVs, a procedure consisting of the hydrolysis of surface-exposed proteins with trypsin (“surface shaving”) was used with a similar methodology to a previously described technique [37] but with some significant modifications. Briefly, *C. glabrata, C. parapsilosis* and *C. tropicalis* EVs were suspended in 400 µL of 25 mM ammonium bicarbonate buffer (NH_4_HCO_3_) with 1 μg of sequencing-grade trypsin (Promega, Madison, WI, USA) and incubated for 10 min at 37 °C. The EVs were then pelleted by ultracentrifugation (as described above) and the collected supernatant was further incubated for 24 h at 37 °C to enhance tryptic digestion. The obtained supernatants were then further centrifuged (15 min, 12,000 *g*), dried in a Speed-Vac (Martin Christ, Osterode am Harz, Germany). The obtained peptides were identified using LC-MS/MS analysis as described earlier [38], being first separated on a 100 mm × 2.1 mm Aeris 3.6 μm PEPTIDE XB-C18 column (Phenomenex, Torrance, CA, USA) with an ultra-high-performance liquid chromatography (LC) Dionex Ultimate 3000 system (Dionex, Sunnyvale, CA, USA), and directly analyzed with LC-coupled HCTUltra ETDII ion-trap mass spectrometer equipped with an electrospray ionization ion source (Bruker, Bremen, Germany). Four independent biological replicates were prepared from the EVs of each investigated *Candida* species.

To also identify the proteins present inside the EVs, the vesicles were suspended in 400 µL of ultrapure water and sonicated twice for 30 s with UP50H Compact Lab Homogenizer (amplitude 80%, cycle 0.5, 50 watts, 30 kHz; Hielscher Ultrasonics, Teltow, Germany). After ultracentrifugation, the protein-containing supernatants were collected and lyophilized in an Alpha 1–2 lyophilizer (Martin Christ). Three independent biological replicates were performed for each *Candida* species. Additionally, membranes that remained from the EVs after sonication and ultracentrifugation were subjected to further protein extraction to enrich the analysis by identifying proteins located within or near the membrane internal or external surface. These pellets were therefore incubated in 200 µL of 25 mM NH_4_HCO_3_ with 0.1 µg of trypsin for 30 min at 37 °C or lyophilized and then further incubated for 30 min with 15 µL of 50 mM Tris-HCl, pH 6.8 supplemented with 0.4% sodium dodecyl sulphate (SDS) and 4% 2-mercaptoethanol (2-ME). After trypsinization, ultracentrifugation was carried out as described above to remove debris and collect peptide-containing supernatant that was subjected to further overnight incubation at 37 °C to enhance tryptic digestion and enable for further LC-MS/MS analysis.

Proteins obtained after lyophilization were separated by SDS-PAGE electrophoresis and stained with Coomassie Brilliant Blue R-250. After obtaining the proteins by EVs sonication, the average amount of protein of 10 µg for *C. glabrata* and *C. tropicalis* and 15 µg for *C. parapsilosis* was applied to the gel per lane. All noticeable protein bands were manually excised; however, if they were smudged, the entire lane was cut into several fragments, then subjected to tryptic digestion in accordance with a procedure described elsewhere [39] and analyzed using LC-MS/MS as described above. The lists of obtained peaks were searched against the nonredundant protein database of the NCBI with taxonomies restricted to fungi (26,490,256 sequences for all entries, 1,924,810 sequences for fungi) using an in-house Mascot server (v.2.3.0; Matrix Science, London, UK). The following search parameters were applied: fixed modification, carbamidomethylation (C); variable modifications, oxidation (M); protein mass, unrestricted; number of missed cleavages, 2; peptide mass tolerance of ± 0.3 Da and fragment mass tolerance of ± 0.5 Da. Proteins with at least one unique peptide and for which the Mascot protein score was higher than 53 were taken into account in further analyzes, and of these proteins, only those that appeared at least twice in all replicates performed were considered the most abundant in the samples tested.

### 2.7. Characterization of C. glabrata, C. tropicalis and C. parapsilosis EVs with High-Resolution Flow Cytometry Analysis

High-resolution flow cytometry analysis with an Apogee A60-Micro-PLUS flow cytometer (Apogee Flow Systems, Hemel Hempstead, UK) was applied for the detection of EV surface-localized α-mannopyranosyl residues with the use of fluorescein-conjugated concanavalin A (Con A; Thermo Fisher Scientific). Prior to use, Con A was centrifuged at 21,000 *g* for 20 min at 4 °C to remove any potential lectin aggregates. Fungal EVs were then suspended in 0.22 μm-filtered PBS (Lonza, Basel, Switzerland) and incubated with Con A (100 µg/mL) for 30 min at room temperature and immediately acquired. Apogee Calibration Beads #1493 and #1517, containing a mixture of green-fluorescent polystyrene (PS) and non-fluorescent silica (Si) beads, were used to calibrate the flow cytometer. The obtained data were analyzed using Apogee Histogram Software and representative plots from two biological replicates for EVs isolated from each *Candida* species were selected for presentation.

## 3. Results

For the preparation of fungal EV samples, *Candida* species were cultured in RPMI 1640 medium. The cultures that were used further for isolation contained approximately 4 × 10^10^ (*C. glabrata* and *C. tropicalis*) or 10 × 10^10^ (*C. parapsilosis*) living cells, which were verified by CFU counting on YPD agar plates. No fungal cells were found in the filtered concentrated supernatants that were subsequently subjected to ultracentrifugation. Nanoparticle tracking analysis (NTA) was used to estimate the number of particles contained in a single EV sample obtained after concentration of the total harvested volume of supernatant from the growth of particular *Candida* species. For *C. glabrata*, the particle number was determined as 2.55 ± 0.17 × 10^10^ of produced EVs, whereas for *C. parapsilosis* this was 2.3 ± 0.08 × 10^10^ particles and for *C. tropicalis* was 8.88 ± 0.63 × 10^9^ particles. The measured contents of proteins and phospholipids per 1 × 10^10^ EVs for each *Candida* species are listed in Table 1.

**Figure 1:**
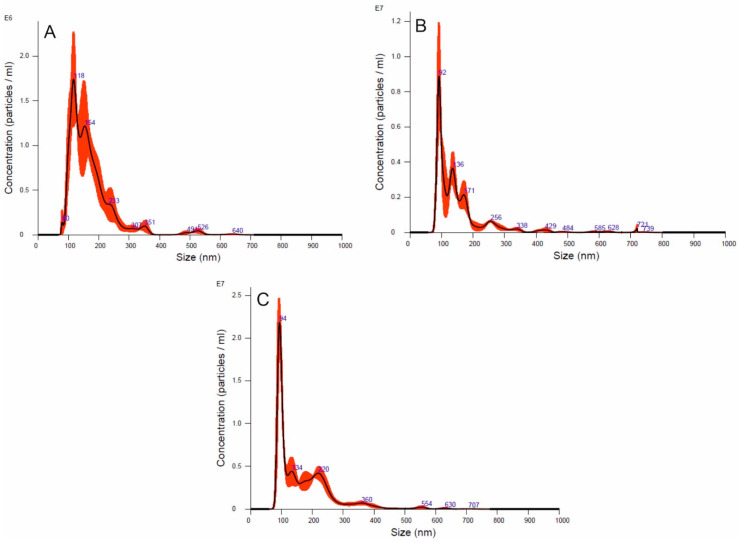
NTA particle size distribution analysis of *C. glabrata* (**A**), *C. parapsilosis* (**B**) and *C. tropicalis* (**C**) EVs. Representative histograms of the average size distribution (black line) from three measurements of a single sample. Red areas specify the standard deviation (SD) between measurements and blue numbers indicate the maxima of individual peaks.

As recommended by the International Society for Extracellular Vesicles [40], the *Candida* EV sizes were measured by NTA and the heterogeneity of the preparations, i.e., the presence of several EV populations of different sizes, was demonstrated. The measurement data are provided in Table 2 and representative histograms for the EV samples from the three *Candida* species under analysis are presented in Figure 1.

**Figure 2:**
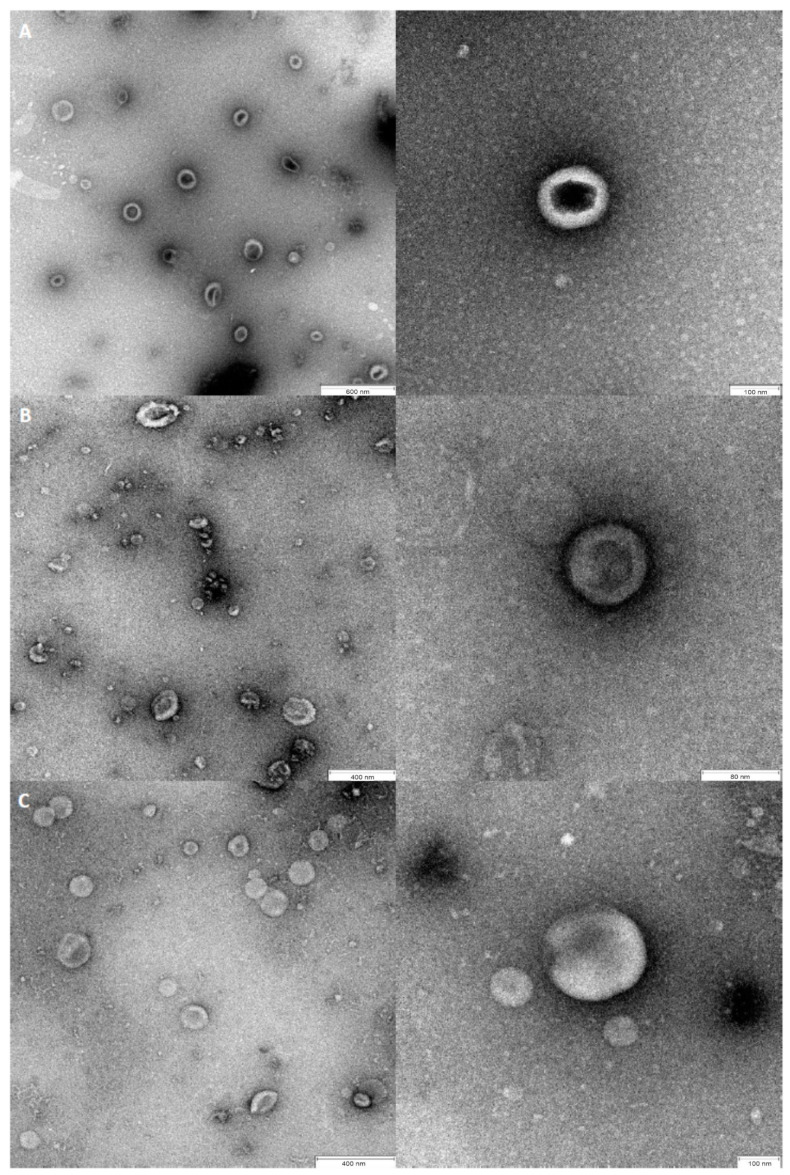
TEM images of isolated *C. glabrata* (**A**), *C. parapsilosis* (**B**) and *C. tropicalis* (**C**) EVs. The nm values of the scale bars are included in each image individually.

Each of the tested *Candida* species produced EVs with sizes ranging from 60–280 nm (90% of total population). The NTA results were confirmed by TEM analysis which revealed spherical structures of different sizes within this size range (Figure 2).

To characterize the protein composition of the EVs, protein identification was performed with LC-MS/MS and the topology of individual molecules was assessed in accordance with ISEV recommendations [40]. EV surface shaving with trypsin was conducted as previously described [41], which is a similar method to that previously performed for whole fungal cells [37,38]. Proteins exposed at the surfaces of *C. glabrata*, *C. parapsilosis* and *C. tropicalis* EVs were then identified. The concentration of trypsin and the reaction time used were selected carefully to avoid breakage of the vesicles and uncontrolled release of their contents [42]. The fungal EVs were also sonicated to disrupt their membrane integrity and enrich for molecules contained within the vesicles. However, several proteins associated with the membrane and cell wall were also identified in this group, indicating that they may become fragmented and detached from the surface during the procedure. Nevertheless, given that transmembrane proteins or proteins anchored in the membrane and thus located in close proximity to it could be difficult to identify using these two abovementioned methods, pellets containing vesicle membrane remnants were collected by ultracentrifugation and subjected to additional hydrolysis with trypsin or protein extraction using detergent and reducing reagent. The lists of the most abundant identified proteins, i.e., those that were identified at least twice within all of the biological replicates analyzed, are presented in Table 3 for *C. glabrata*, Table 4 for *C. parapsilosis* and Table 5 for *C. tropicalis* EVs. Additionally, all proteins that were identified in all biological replicates and those that were identified only once in a particular fraction are listed in Supplementary Tables S1–S3, together with the raw data and mass spectrometry identification parameters.

We identified 42 highly abundant EV proteins from *C. glabrata*, 33 from *C. parapsilosis*, and 34 from *C. tropicalis*. We found that 32, 22 and 30 of these abundant proteins in *C. glabrata*, *C. parapsilosis* and *C. tropicalis* EVs, respectively, were surface-exposed proteins. In addition, we identified three new proteins for *C. glabrata* and *C. tropicalis*, and 10 for *C. parapsilosis* after sonication. Extending the procedure by further additional step allowed for identification of seven membrane proteins from *C. glabrata* EVs and one cell wall protein for the other two species, i.e., a *C. parapsilosis* putative GPI-anchored protein (Pga17) and a *C. tropicalis* putative adhesin-like cell wall mannoprotein CTRG_02188 (Flo9). This number of identified proteins was lower than in some other proteomic studies on fungal EVs probably due to the differences in the equipment used and detailed methodology that allowed to identify only those proteins that were most abundant in the sample. However, some contamination of individual fractions with proteins from other fractions, which may have various causes, cannot be excluded; therefore particular caution is needed in the interpretation of results that are essentially a preliminary indication of the potential location of these proteins.

**Figure 3:**
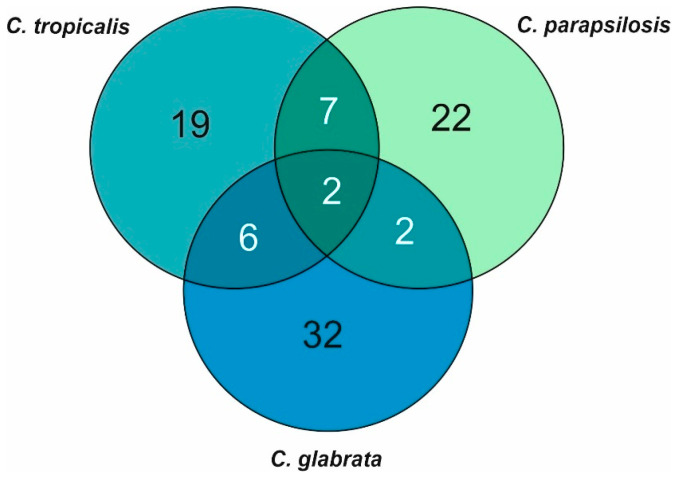
Numbers of specific and shared proteins among *C. glabrata*, *C. parapsilosis* and *C. tropicalis* EVs.

The Venn diagram shown in Figure 3 indicates that although most of the abundant EV proteins were specific to that *Candida* species, several EV-derived proteins were shared between species, and two of these proteins—cell wall protein Scw4 and alcohol dehydrogenase Adh1―were common to *C. glabrata*, *C. parapsilosis* and *C. tropicalis* EVs. The proteins found in the EVs from *C. glabrata* and *C. parapsilosis* included exo-1,3-beta-glucanase (Exg1/Xog1) and GPI-anchored cell wall protein (Ecm33). Seven proteins were shared for *C. tropicalis* and *C. parapsilosis* EVs, including two glycosidases (Phr1 and 2), 1,3-beta-glucosyltransferase (Bgl2) and cell wall mannoprotein (Mp65). *C. tropicalis* and *C. glabrata* EVs shared six proteins, including enolase (Eno1), glyceraldehyde-3-phosphate dehydrogenase (Tdh3), phosphoglycerate kinase (Pgk1) and pyruvate kinase (Cdc19), which are highly evolutionarily conserved and common proteins between different cells, hence the most likely their sharing between less related species.

**Figure 4:**
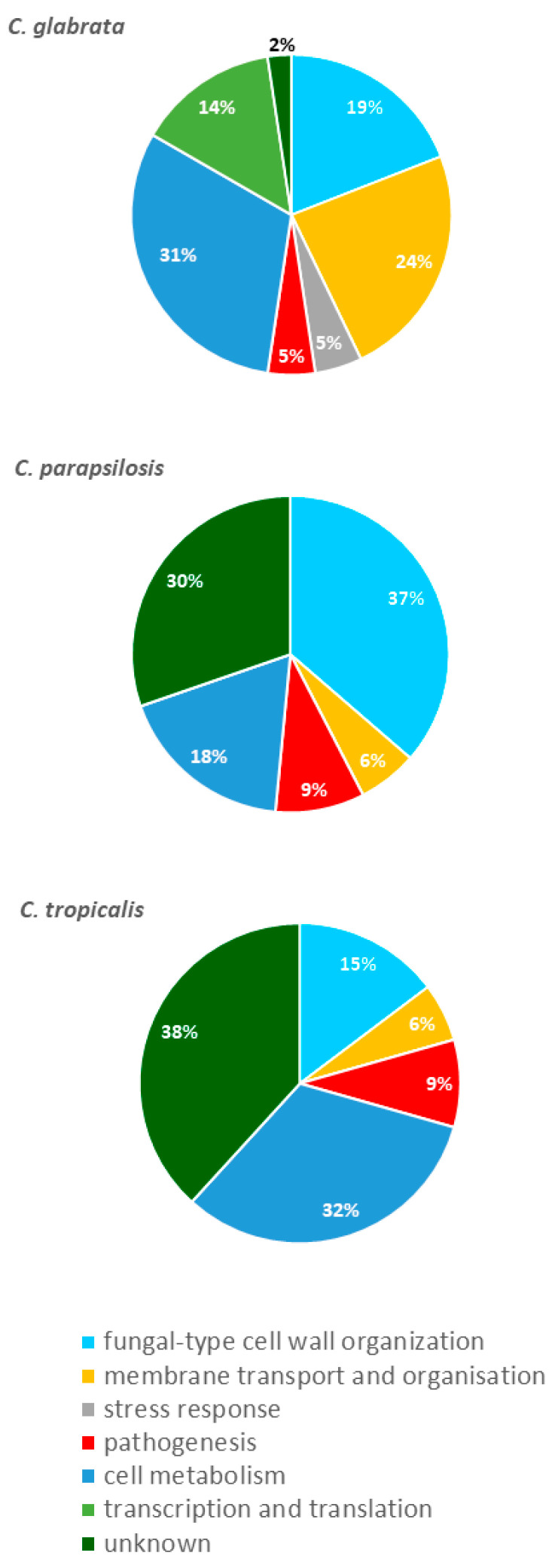
Distribution of EV proteins in *C. glabrata*, *C. parapsilosis* and *C. tropicalis* within major functional groups for similar cellular processes.

The protein descriptions and gene ontology (GO) annotations for each of *C. glabrata*, *C. parapsilosis* or *C. tropicalis* orthologous genes were assigned based on the information from the Candida Genome Database (CGD, http://www.candidagenome.org) [43], Saccharomyces Genome Database (SGD, https://www.yeastgenome.org) [44] and UniProtKB Database (https://www.uniprot.org) [45]. Proteins identified in the EVs of individual non-albicans *Candida* species were classified into functional groups according to the appropriate GO annotations and with regard to their principal biological functions. Those groups were as follows: (i) fungal-type cell wall organization, (ii) membrane transport and organization, (iii) stress response, (iv) pathogenesis, (v) cell metabolism, (vi) transcription and translation, and (vii) proteins of unknown function. A graphic representation of this functional division is presented in Figure 4 and indicates that the largest functional group of EV proteins, other than proteins of unknown function, was cell metabolism for *C. glabrata* (31%) and *C. tropicalis* (32%) and cell wall organization for *C. parapsilosis* (37%). The second largest group included proteins involved in membrane transport and organization for *C. glabrata* (24%), cell metabolism for *C. parapsilosis* (18%) and cell wall organization for *C. tropicalis* (14%). In the case of *C. glabrata*, EV proteins involved in translation (14%) were significantly represented.

**Figure 5:**
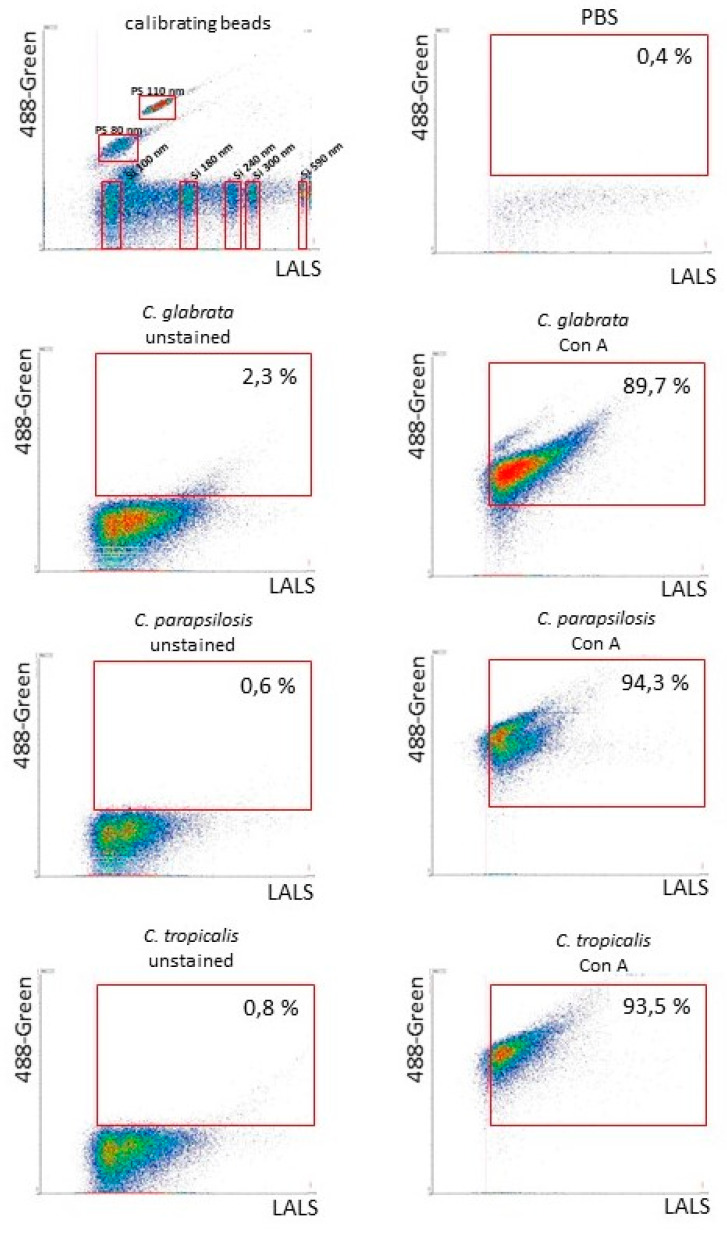
High-resolution flow cytometric detection of EV surface mannoproteins from *C. glabrata*, *C. parapsilosis* and *C. tropicalis*. Samples were analyzed with an Apogee A60-Micro-PLUS flow cytometer dedicated to the analysis of small particles. The dot plot for calibrating beads #1493 and #1517, containing a mixture of green-fluorescent polystyrene (PS) and non-fluorescent silica (Si) beads, is included as a size distribution reference. Representative dot plots of fungal EVs unstained and stained with concanavalin A (Con A) conjugated with fluorescein are presented. The percentage of objects positive for Con A binding is shown in selected regions marked with red frames. LALS, large angle light scatter parameter, proportional to the size of acquired objects.

High-resolution flow cytometry was next used for the detection of α-mannopyranosyl residues within EV surface exposed glycoproteins with the use of fluorescein-conjugated Con A, a lectin that recognizes mannose-containing glycoconjugates (Figure 5). Calibration of the flow cytometer using appropriately sized beads was performed and it was verified that the PBS buffer used for the dilution of EV preparations did not contain significant numbers of particles. Upon comparing the fluorescence intensity of unstained vesicles with that of Con A-fluorescein-bound vesicles, the exposure of mannoproteins on the EV surface was confirmed for each of the *Candida* species tested. The percentage of positive objects in this regard was close to 90% in the case of *C. glabrata*, and greater than 90% for *C. parapsilosis* and *C. tropicalis*.

## 4. Discussion

EVs are ubiquitously produced by organisms of all kingdoms and take part in important biological processes in cells, in intercellular communication, and also in interactions between pathogens and their host during infection [46]. These bodies are richly loaded with variety of cargos that are protected by a lipid bilayer and transported from the cell to the external milieu through different mechanisms which are not yet fully recognized. Vesicle populations are often heterogenous in size and composition, depending on the secretion pathway, the physiological state of the cells, or the influence of the extracellular environment, and this is reflected in the large variety of their functions [47]. The crucial role of EVs in health disorders and infectious diseases has led to a continuous and increasing interest in these structures [46]. The composition and role of EVs have been characterized to some extent for several species of fungi that are pathogenic to humans [23,24,25,28]. Among *Candida* species, however, such data are currently available only for the EVs produced by *C. albicans* [26,27,30,34,48]. Imaging results for these extracellular structures have been published for *C. parapsilosis* [22].

In our present study, we have for the first time characterized the EVs produced by *C. glabrata*, *C. parapsilosis* and *C. tropicalis*, with a particular emphasis on the vesicular proteome. The EVs produced by planktonic cells were isolated in our experiments but the fungi were not grown in the YPD medium routinely used for yeast culture but in buffered RPMI 1640 medium that provides all the components necessary for growth, but without proteinaceous content. The number of vesicles produced by fungal cells under these conditions was then determined, although it was lower than the number of living cells in each case which was probably due to the general difficulties in multi-stage EV isolation procedures using culture supernatants. The phospholipid and protein contents were also measured and clearly revealed major vesicle-building components in the obtained preparations. The differences between the protein concentration for EVs of individual species were not statistically significant, whereas the highest lipid concentration was demonstrated for *C. parapsilosis* EVs, with the lowest found for *C. glabrata*.

Analysis of the sizes of the isolated *C. glabrata*, *C. parapsilosis* or *C. tropicalis* EVs using NTA and TEM revealed heterogeneous populations for each tested species. This is also the case in the vesicles produced by the planktonic form of *C. albicans* in which fractions of EVs of 50–100 nm and larger than 200 nm in size were previously described [26,27].

Mass spectrometry identification of proteins from *C. glabrata*, *C. parapsilosis* and *C. tropicalis* vesicles revealed the presence of numerous proteins involved in the membrane transport of different molecules including glucose, siderophores, copper and hydrogen ions, suggesting a plasma membrane origin of the EVs. This group of proteins was particularly represented in the case of the *C. glabrata* EVs, where only in the fraction of membrane-associated proteins Hxt5 and Hxt6, Put4, Ctr1, Arn1 and Tna1 were identified. In addition, the proteins involved in the building, remodelling and integrity of the fungal cell wall were abundant in EVs from all three tested species. Cell wall glucanases, including Eng1, Exg1/Xog1, Bgl2 and Scw4 were indicated to be EV-derived proteins shared between these investigated NAC organisms. This last protein was identified for all three investigated species both at the surface and in the inside of the EVs, similarly to the location of Bgl2 for *C. parapsilosis* and *C. tropicalis*. The activity of these factors may be important in the process of vesicle passage through the cell wall to the external environment, but also may play a role in the remodelling of surface polysaccharides, enabling fungi to modulate their interactions with host immune cells by interfering with the recognition of pathogens via specific receptors [49]. The typical candidal cell wall proteins are most often highly glycosylated and equipped with branched O- and N-linked mannosides [50]. Their presence at the surface of *C. glabrata*, *C. parapsilosis* and *C. tropicalis* vesicles was also confirmed in our current experiments by high resolution flow cytometry after their staining with the ConA lectin that recognizes mannose residues, thus corroborating the identification of several mannoproteins in EVs using LC-MS/MS.

Regarding the postulated significant role of EVs in the virulence of *Candida* fungi, which has been previously suggested for *C. albicans* [27,34], it was particularly important in our present analyses to identify the proteinaceous surface components of EVs that would be capable of mediating their immediate and direct contact with host proteins and cells during infection. Some of the vesicular proteins identified in our present analysis have a reported role in adhesion and pathogenesis that was directly attributed previously based on the functions of their orthologous proteins. These include the *C. parapsilosis* yeast cell wall protein Ywp1 involved in biofilm dispersal and β-1,3-glucan masking in yeast cells [51,52], the *C. parapsilosis* and *C. tropicalis* cell wall mannoprotein Mp65 that exhibits adhesive properties and contributes to fungal virulence and the stimulation of dendritic cells [53,54] and inducible acid phosphatase Pho100 [55], which has also been identified at the EV surface in these two *Candida* species. Mp65 was also identified inside the EVs of these two species. In addition, two aspartic proteinases belonging to larger families of proteins involved in proteolysis and virulence―*C. glabrata* yapsin Yap3 and *C. tropicalis* Sapt4―were also identified in our analysis, although their specific role in infections requires further study [56,57,58]. *C. glabrata* proteinase was identified in all tested fractions isolated during EVs analysis, whereas the enzyme of *C. tropicalis* only at their surface. For some of the typical proteins associated with the fungal cell wall and identified in the EVs from our current NAC species, and additional involvement in pathogenesis apart from cell wall organization was also indicated in prior reports for their orthologs, such as cell wall 1,3-beta-glucosyltransferase Bgl2 [59] and glycosidase Phr 2 [60] that we here identified from the *C. parapsilosis* and *C. tropicalis* vesicles.

A large proportion of the proteins we identified in the EVs produced by each of the three NAC species in our current study are involved in basic cell metabolism. This may indicate that these vesicles are derived from the cytoplasm and thus carry many intracellular enzymes [18]. As such proteins are often found outside of fungal cells or at their surface [37] but do not possess a signal sequence directing them to a classical secretory pathway, there is likely to be another unconventional pathway leading to their secretion, i.e., EV transport [61]. Numerous cytoplasmic proteins have already been identified in *C. albicans* vesicles, including enolase (Eno1), alcohol dehydrogenase (Adh1), phosphoglycerate kinase (Pgk1) and glyceraldehyde-3-phosphate dehydrogenase (Tdh3) [62]. In the case of the EVs from our current NAC species, the cytoplasmic proteins we identified included Adh1, Eno1, Pgk1, Tdh3, pyruvate kinase Cdc19, pyruvate decarboxylase Pdc1 and 6-phosphogluconate dehydrogenase Gnd1. In the case of all these proteins identified for *C. glabrata*, they were found in all three analyzed fractions, both at the surface of the vesicles and inside them, whereas in the case of *C. tropicalis*, the surface location predominated for most of them among the proteins identified for this species, except for Eno1 and Pgk1 also found inside of the vesicles. Although these proteins have a cytoplasmic origin and are involved in basic metabolism in accordance with their primary function, important roles in host–pathogen interactions have been repeatedly attributed to these factors or their orthologs in *C. albicans*, thus defining them as moonlighting proteins and candidal molecules contributing to virulence [63,64,65]. *C. tropicalis* enolase was identified previously as a protein that binds human high-molecular-mass kininogen, a component of the plasma kinin-generating system involved in innate immunity, and human vitronectin, fibronectin and laminin [66,67]. It was also demonstrated in our current study that Eno1 is present at the surface of *C. tropicalis* and *C. glabrata* EVs. Adh1 was identified in the EVs of all three investigated NAC species in our present study. Its ortholog from *C. albicans* is known to be immunogenic during infection and is responsible for binding of extracellular matrix proteins and for the differentiation of human monocytes to macrophages enhancing their adhesion, phagocytosis, and killing capacities [68,69,70]. In addition, other proteins identified in fungal vesicles might be immunogenic during infection, including *C. parapsilosis* Adh1 [71] and *C. tropicalis* Eno1 and Tdh3 [72], thus being potential candidates for future immunodiagnostic tests or vaccines.

Of the EV proteins identified for *Candida* species, many of them were also indicated in the extracellular vesicles of other fungi, both *S. cerevisiae* and other species pathogenic to humans [21]. Such intracellular proteins as Pgk1, Tdh3 and Gnd1 were also found in EVs of *P. brasiliensis*, *H. capsulatum*, *C. neoformans* or *S. cerevisiae*, Eno1 was identified for *C. neoformans*, *P. brasiliensis* and *S. cerevisiae*, and Fba1 for the last two species and *H. capsulatum* [12,22,23,73]. This indicates a fairly common phenomenon associated with the presence of these proteins in the EVs among different fungal organisms, which may be associated with their unusual secretion. In addition, among the proteins involved in transmembrane transport, two, i.e., ADP, ATP carrier protein and plasma membrane ATPase identified for *C. glabrata* or *C. tropicalis* were also found in EVs of *C. neoformans*, *P. brasiliensis*, *H. capsulatum* and *S. cerevisiae* [12,22,23,73] and enzymes responsible for cell wall remodelling such as Exg1 and Scw4 or cell wall associated protein Ecm33 were also found in *S. cerevisiae* vesicles [73].

The characteristics of EVs produced by *C. glabrata*, *C. parapsilosis* and *C. tropicalis* presented in our current analysis significantly contribute to our understanding of the biology of these pathogens, and the importance of these vesicles in the pathogenesis of infections caused by NAC species. It is particularly noteworthy in this regard that these structures are involved in the transport of a number of fungal virulence factors.

## Figures and Tables

**Table 1:** Average protein and lipid contents in EVs obtained from *C. glabrata*, *C. parapsilosis* and *C. tropicalis*.

	Protein Content (Micrograms per 1 × 10^10^ Vesicles)	Phospholipid Content (Nanomoles of Lecithin Equivalents per 1 × 10^10^ Vesicles)
*C. glabrata*	4.69 ± 0.87	4.79 ± 0.61
*C. parapsilosis*	10.02 ± 3.99	17.67 ± 3.8
*C. tropicalis*	8.15 ± 3.5	10.32 ± 0.22

**Table 2:** Average EV sizes measured by NTA. Parameters D10, D50 and D90 indicate that 10%, 50% or 90% of the EV population, respectively, had a diameter of less than or equal to the specified value. Data are presented as means ± SEM.

	Mean (nm)	Mode (nm)	SD (nm)	D10 (nm)	D50 (nm)	D90 (nm)
*C. glabrata*	171.0 ± 1.6	139.5 ± 7.7	74.9 ± 0.8	100.2 ± 5.65	157.9 ± 4.3	254.5 ± 10.5
*C. parapsilosis*	128.5 ± 25.2	84.65 ± 11.65	68.95 ± 24.15	78.9 ± 10.06	107.3 ± 21.55	203.5 ± 52.4
*C. tropicalis*	148.2 ± 21.75	90.65 ± 3.05	75.35 ± 18.15	83.7 ± 6.7	125.7 ± 17.1	242.3 ± 35.55

**Table 3:** Mass spectrometry identification of abundant proteins in *C. glabrata* EVs after vesicle surface shaving with trypsin (T) or vesicle sonication (S), and in EV membrane protein-enriched fractions (M). *The peptides obtained were analyzed using the Dionex Ultimate 3000 UHPLC system coupled to an HCTUltra ETDII mass spectrometer and the obtained lists of peaks were searched against the NCBI protein database using an in-house Mascot server.*

NCBI Accession Number	Protein Description	T	S	M
gi|18073449 (CAC83344)	GAS-1 homologue	+	+	
gi|20258069 (AAM16160)	phospholipase B		+	
gi|302309608 (XP_444845)	CAGL0A01826g probable glucose transporter (Hxt5)			+
gi|50284733 (XP_444794)	CAGL0A00495g plasma membrane ATPase 1 (Pma1)		+	+
gi|50284865 (XP_444860)	CAGL0A02211g high-affinity hexose transporter (Hxt7)	+		+
gi|50284867 (XP_444861)	CAGL0A02233g high-affinity hexose transporter (Hxt6)			+
gi|50284869 (XP_444862)	CAGL0A02255g uncharacterized protein	+		+
gi|50284959 (XP_444908)	CAGL0A03234g elongation factor 2 (Eft2)	+		+
gi|50285099 (XP_444978)	CAGL0A04829g hexokinase (Hxk2)	+		
gi|50285355 (XP_445106)	CAGL0B03069g transaldolase (Tal1)	+		
gi|50286153 (XP_445505)	CAGL0D02090g GDP-dissociation inhibitor (Asc1)	+		
gi|50286375 (XP_445616)	CAGL0D04708g copper transport protein (Ctr1)			+
gi|50286669 (XP_445764)	CAGL0E01727g putative aspartic protease (Yap3)	+	+	+
gi|50286871 (XP_445865)	CAGL0E04092g siderophore iron transporter (Arn1)			+
gi|50287007 (XP_445933)	AGL0E05632g proline and gamma-aminobutyrate permease (Put4)			+
gi|50287107 (XP_445983)	CAGL0F00209g high-affinity nicotinic acid transporter (Tna1)			+
gi|50287735 (XP_446297)	CAGL0F07579g cell wall mannoprotein (Cwp1)	+	+	+
gi|50287897 (XP_446378)	CAGL0G00308g cell wall protein with similarity to glucanases (Scw4)	+	+	+
gi|50287951 (XP_446404)	60S acidic ribosomal protein P0	+	+	
gi|50288681 (XP_446770)	CAGL0G09383g glyceraldehyde-3-phosphate dehydrogenase 3 (Tdh3)	+	+	+
gi|50288687 (XP_446773)	CAGL0G09515g sporulation-specific exo-1,3-beta-glucanase (I/II) (Exg1/Spr1)	+	+	+
gi|50289283 (XP_447072)	CAGL0H06369g cystathionine gamma-lyase (Cys3)	+		+
gi|50289307 (XP_447084)	CAGL0H06633g phosphoenolpyruvate carboxykinase (Pck1)	+		
gi|50289515 (XP_447189)	60S ribosomal protein L1	+		
gi|50289685 (XP_447274)	CAGL0I00484g exo-1,3-beta-glucanase (Exg1)	+	+	+
gi|50289857 (XP_447360)	CAGL0I02486g enolase I (Eno1)	+	+	+
gi|50290013 (XP_447438)	CAGL0I04356g translation initiation factor eIF4A (Tif1)	+	+	+
gi|50290317 (XP_447590)	CAGL0I07843g alcohol dehydrogenase I (Adh1)	+	+	+
gi|50291073 (XP_447969)	CAGL0J06050g secreted glycoprotein (Ygp1)	+	+	
gi|50292035 (XP_448450)	CAGL0K05137g vacuolar acid trehalase precursor (Ath1)	+	+	+
gi|50292597 (XP_448731)	CAGL0K11858g putative flavodoxin (Pst2)	+		+
gi|50292725 (XP_448795)	CAGL0L00495g heat shock protein (Hsc82)	+		
gi|50292739 (XP_448802)	acetate-CoA ligase (Acs1)	+	+	+
gi|50292893 (XP_448879)	CAGL0L02497g fructose-bisphosphate aldolase (Fba1)	+		+
gi|50293403 (XP_449113)	CAGL0L07722g phosphoglycerate kinase (Pgk1)	+	+	+
gi|50293465 (XP_449144)	CAGL0L08448g non-classical export protein 2 (Nce102)			+
gi|50294025 (XP_449424)	CAGL0M01826g GPI-anchored protein (Ecm33)	+	+	
gi|50294171 (XP_449497)	CAGL0M03465g ammonia transport outward protein 1 (Spg2)	+	+	
gi|50294560 (XP_449691)	CAGL0M07920g pyruvate decarboxylase (Pdc1)	+	+	+
gi|50294908 (XP_449865)	CAGL0M12034g pyruvate kinase (Cdc19)	+	+	+
gi|50295024 (XP_449923)	CAGL0M13343g 6-phosphogluconate dehydrogenase (Gnd1)	+	+	+
gi|50295070 (XP_449946)	CAGL0M13849g glycophospholipid-anchored surface glycoprotein (Gas2)		+	

**Table 4:** Mass spectrometry identification of abundant proteins in *C. parapsilosis* EVs after vesicle surface shaving with trypsin (T) or vesicle sonication (S), and in EV membrane protein-enriched fractions (M). *The resulting peptides were analyzed using the Dionex Ultimate 3000 UHPLC system coupled to an HCTUltra ETDII mass spectrometer and the obtained lists of peaks were searched against the NCBI protein database using an in-house Mascot server.*

NCBI Accession Number	Protein Description	T	S	M
gi|21953342 (CAC86400)	lipase 2 (Lip2)		+	
gi|354543255 (CCE39973)	hypothetical protein CPAR2_100110 GPI-anchored cell surface protein (Pga4)		+	
gi|354543404 (CCE40123)	hypothetical protein CPAR2_101610 putative aminopeptidase yscI precursor (Lap41)		+	
gi|354543610 (CCE40331)	hypothetical protein CPAR2_103690 cell wall protein (Pga45)	+	+	
gi|354543842 (CCE40564)	hypothetical protein CPAR2_106000 exo-1,3-beta-glucanase (Xog1)	+		
gi|354543893 (CCE40615)	hypothetical protein CPAR2_106500 putative cell wall protein (Scw4)	+	+	+
gi|354543976 (CCE40698)	hypothetical protein CPAR2_107330	+	+	+
gi|354543994 (CCE40716)	hypothetical protein CPAR2_107510 putative carboxypeptidase Y precursor (Prc3)	+		
gi|354544089 (CCE40811)	hypothetical protein CPAR2_108490	+		
gi|354544096 (CCE40818)	hypothetical protein CPAR2_108560 GPI-anchored cell wall protein (Ecm33)	+	+	+
gi|354544127 (CCE40850)	hypothetical protein CPAR2_108890 glucan endo-1,3-beta-D-glucosidase (Eng1)		+	
gi|354544206 (CCE40929)	hypothetical protein CPAR2_109660 glycosidase (Phr2)	+	+	
gi|354544345 (CCE41068)	hypothetical protein CPAR2_300570	+	+	
gi|354544403 (CCE41126)	hypothetical protein CPAR2_301150 predicted membrane protein induced during mating (Fmp45)		+	+
gi|354544501 (CCE41225)	hypothetical protein CPAR2_302140 cell surface glycosidase (Phr1)		+	
gi|354544883 (CCE41608)	hypothetical protein CPAR2_801600 subtilisin-like protease (proprotein convertase) (Kex2)	+		
gi|354544910 (CCE41635)	hypothetical protein CPAR2_801850 Ala- Leu- and Ser-rich protein (Op4)	+	+	+
gi|354545228 (CCE41955)	hypothetical protein CPAR2_805040 putative adhesin-like	+	+	+
gi|354545372 (CCE42100)	hypothetical protein CPAR2_806490 1,3-beta-glucan-linked structural cell wall protein (Pir1)	+	+	+
gi|354545390 (CCE42118)	hypothetical protein CPAR2_806670 secreted yeast cell wall protein (Ywp1)	+	+	
gi|354545518 (CCE42246)	hypothetical protein CPAR2_807950	+	+	+
gi|354546478 (CCE43208)	hypothetical protein CPAR2_208530 putative inducible acid phosphatase (Pho100)	+		+
gi|354546810 (CCE43542)	hypothetical protein CPAR2_211860 putative GPI-anchored protein (Pga17)			+
gi|354547091 (CCE43824)	secreted hypothetical protein CPAR2_500500	+	+	+
gi|354547255 (CCE43989)	hypothetical protein CPAR2_502140 GPI-linked chitinase (Cht2)		+	
gi|354547299 (CCE44033)	hypothetical protein CPAR2_502580 alcohol dehydrogenase (Adh1)		+	
gi|354547623 (CCE44358)	hypothetical protein CPAR2_401600 1,3-beta-glucosyltransferase, cell wall enzyme (Bgl2)	+	+	+
gi|354547664 (CCE44399)	hypothetical protein CPAR2_402000 GPI-anchored protein of cell wall (Pga30)		+	
gi|354548052 (CCE44788)	hypothetical protein CPAR2_405910 vacuolar membrane protein (Abg1)	+		+
gi|354548190 (CCE44926)	hypothetical protein CPAR2_407280	+	+	+
gi|354548203 (CCE44939)	hypothetical protein CPAR2_407410 cell surface mannoprotein (Mp65)	+	+	+
gi|354548325 (CCE45061)	hypothetical protein CPAR2_700650 Pry family cell wall protein (Rbe1)		+	
gi|354548638 (CCE45375)	hypothetical protein CPAR2_703880 putative mannosyltransferase (Mnn7)	+		

**Table 5:** Mass spectrometry identification of abundant proteins in *C. tropicalis* EVs after vesicle surface shaving with trypsin (T) or vesicle sonication (S), and in EV membrane protein-enriched fractions (M). *The resulting peptides were analyzed using the Dionex Ultimate 3000 UHPLC system coupled to an HCTUltra ETDII mass spectrometer and the obtained lists of peaks were searched against the NCBI protein database using an in-house Mascot server.*

NCBI Accession Number	Protein Description	T	S	M
gi|220900339 (ACL82370)	secreted aspartyl protease (Sapt4)	+		
gi|255720907 (XP_002545388)	cell wall glucan 1,3-beta-glucosidase precursor CTRG_00169 (Bgl2)	+	+	
gi|255721523 (XP_002545696)	cell wall protein with similarity to Hwp1 CTRG_00477 (Rbt1)	+	+	
gi|255722347 (XP_002546108)	putative constitutive acid phosphatase CTRG_00890 (Pho113)	+		
gi|255722852 (XP_002546360)	putative GPI-anchored adhesin-like protein CTRG_05838 (Hyr3)	+	+	+
gi|255722954 (XP_002546411)	lysophospholipase 1 precursor CTRG_05889 (Plb3)	+		
gi|255723403 (XP_002546635)	alcohol dehydrogenase 1 CTRG_06113 (Adh1)	+		
gi|255723898 (XP_002546878)	putative GPI-anchored protein CTRG_01183 (Pga17)	+	+	
gi|255724450 (XP_002547154)	pyruvate kinase CTRG_01460 (Cdc19)	+		
gi|255725506 (XP_002547682)	protein of unknown function CTRG_01989	+	+	+
gi|255725714 (XP_002547786)	cell surface mannoprotein CTRG_02093 (Mp65)	+	+	
gi|255725930 (XP_002547891)	putative adhesin-like cell wall mannoprotein CTRG_02188 (Flo9)			+
gi|255727360 (XP_002548606)	secreted protein CTRG_02903	+		
gi|255727428 (XP_002548640)	phosphoglycerate kinase CTRG_02937 (Pgk1)	+	+	
gi|255727881 (XP_002548866)	enolase 1 CTRG_03163 (Eno1)	+	+	
gi|255728149 (XP_002549000)	extracellular/plasma membrane-associated glucoamylase CTRG_03297 (Gca1)	+		
gi|255728237 (XP_002549044)	putative adhesin-like protein CTRG_03341	+	+	
gi|255728723 (XP_002549287)	opaque-phase-specific protein OP4 precursor CTRG_03584 (Op4)	+	+	+
gi|255729274 (XP_002549562)	secreted protein CTRG_03859	+	+	+
gi|255729440 (XP_002549645)	cell surface glycosidase CTRG_03942 (Phr1)	+		
gi|255729820 (XP_002549835)	putative inducible acid phosphatase CTRG_04132 (Pho100)	+	+	
gi|255729832 (XP_002549841)	conserved hypothetical protein CTRG_04138	+	+	
gi|255729942 (XP_002549896)	cell wall acid trehalase CTRG_04193 (Atc1)	+	+	
gi|255730149 (XP_002549999)	glycosidase CTRG_04296 (Phr2)	+		
gi|255730873 (XP_002550361)	putative cell wall protein CTRG_04659 (Scw4)	+	+	
gi|255731107 (XP_002550478)	predicted protein CTRG_04776		+	
gi|255731223 (XP_002550536)	protein of unknown function CTRG_04834	+	+	+
gi|255731592 (XP_002550720)	peptidyl-prolyl cis-trans isomerase CTRG_05018 (Cyp1)	+		
gi|255732093 (XP_002550970)	ADP,ATP carrier protein CTRG_05268		+	
gi|255732521 (XP_002551184)	alcohol dehydrogenase 2 CTRG_05482 (Adh2)	+	+	+
gi|255732780 (XP_002551313)	putative plasma membrane protein CTRG_05611		+	+
gi|255732890 (XP_002551368)	glyceraldehyde-3-phosphate dehydrogenase CTRG_05666 (Tdh3)	+		
gi|255732910 (XP_002551378)	predicted protein CTRG_05676	+		
gi|255733002 (XP_002551424)	plasma membrane H(+)-ATPase CTRG_05722 (Pma1)	+	+	

## Supplementary Materials

The following are available online at https://www.mdpi.com/2073-4409/9/7/1722/s1, Supplementary Table S1. Mass spectrometry identification of *C. glabrata* proteins in EVs after vesicle surface shaving with trypsin, vesicle sonication, or the preparation of fractions enriched with membrane proteins. The resulting peptides were analyzed using the Dionex Ultimate 3000 UHPLC system coupled to an HCTUltra ETDII mass spectrometer and the obtained lists of peaks were searched against the NCBI protein database using an in-house Mascot server. Supplementary Table S2. Mass spectrometry identification of *C. parapsilosis* proteins in EVs after vesicle surface shaving with trypsin, vesicle sonication, or the preparation of fractions enriched with membrane proteins. The resulting peptides were analyzed using the Dionex Ultimate 3000 UHPLC system coupled to an HCTUltra ETDII mass spectrometer and the obtained lists of peaks were searched against the NCBI protein database using an in-house Mascot server. Supplementary Table S3. Mass spectrometry identification of *C. tropicalis* proteins in EVs after vesicle surface shaving with trypsin, vesicle sonication, or the preparation of fractions enriched with membrane proteins. The resulting peptides were analyzed using the Dionex Ultimate 3000 UHPLC system coupled to an HCTUltra ETDII mass spectrometer and the obtained lists of peaks were searched against the NCBI protein database using an in-house Mascot server.

## References

[1] Lamoth F., Lockhart S.R., Berkow E.L., Calandra T. (2018). Changes in the epidemiological landscape of invasive candidiasis. J. Antimicrob. Chemother..

[2] Quindós G., Marcos-Arias C., San-Millán R., Mateo E., Eraso E. (2018). The continuous changes in the aetiology and epidemiology of invasive candidiasis: From familiar *Candida albicans* to multiresistant *Candida auris*. Int. Microbiol..

[3] Lee W.J., Hsu J.F., Lai M.Y., Chiang M.C., Lin H.C., Huang H.R., Wu I.H., Chu S.M., Fu R.H., Tsai M.H. (2018). Factors and outcomes associated with candidemia caused by non-albicans *Candida* spp versus *Candida albicans* in children. Am. J. Infect. Control..

[4] Kumar J., Eilertson B., Cadnum J.L., Whitlow C.S., Jencson A.L., Safdar N., Krein S.L., Tanner W.D., Mayer J., Samore M.H. (2019). Environmental contamination with *Candida* species in multiple hospitals in a Tertiary Care Hospital with a *Candida auris* outbreak. Pathog. Immun..

[5] Orsetti E., Brescini L., Mazzanti S., Trave F., Morroni G., Masucci A., Barchiesi F. (2019). Characterisation of candidemia in patients with recent surgery: A 7-year experience. Mycoses.

[6] Pfaller M.A., Diekema D.J., Turnidge J.D., Castanheira M., Jones R.N. (2019). Twenty Years of the SENTRY Antifungal Surveillance Program: Results for *Candida* Species From 1997–2016. Open Forum Infect. Dis..

[7] Mohr A., Simon M., Joha T., Hanses F., Salzberger B., Hitzenbichler F. (2020). Epidemiology of candidemia and impact of infectious disease consultation on survival and care. Infection.

[8] Enoch D.A., Yang H., Aliyu S.H., Micallef C. (2017). The Changing Epidemiology of Invasive Fungal Infections. Methods Mol. Biol..

[9] Giacobbe D.R., Maraolo A.E., Simeon V., Magnè F., Pace M.C., Gentile I., Chiodini P., Viscoli C., Sanguinetti M., Mikulska M. (2020). Changes in the relative prevalence of candidemia due to non-albicans *Candida* species in adult in-patients: A systematic review, meta-analysis, and meta-regression. Mycoses.

[10] Mesini A., Mikulska M., Giacobbe D.R., Del Puente F., Gandolfo N., Codda G., Orsi A., Tassinari F., Beltramini S., Marchese A. (2020). Changing epidemiology of candidemia: Increase in fluconazole-resistant *Candida parapsilosis*. Mycoses.

[11] Eisenman H.C., Frases S., Nicola A.M., Rodrigues M.L., Casadevall A. (2009). Vesicle-associated melanization in *Cryptococcus neoformans*. Microbiology.

[12] Vallejo M.C., Nakayasu E.S., Matsuo A.L., Sobreira T.J., Longo L.V., Ganiko L., Almeida I.C., Puccia R. (2012). Vesicle and vesicle-free extracellular proteome of *Paracoccidioides brasiliensis*: Comparative analysis with other pathogenic fungi. J. Proteome Res..

[13] Wolf J.M., Espadas-Moreno J., Luque-Garcia J.L., Casadevall A. (2014). Interaction of *Cryptococcus neoformans* extracellular vesicles with the cell wall. Eukaryot Cell.

[14] Peres da Silva R., Puccia R., Rodrigues M.L., Oliveira D.L., Joffe L.S., César G.V., Nimrichter L., Goldenberg S., Alves L.R. (2015). Extracellular vesicle-mediated export of fungal RNA. Sci. Rep..

[15] Zhao K., Bleackley M., Chisanga D., Gangoda L., Fonseka P., Liem M., Kalra H., Al Saffar H., Keerthikumar S., Ang C.S. (2019). Extracellular vesicles secreted by *Saccharomyces cerevisiae* are involved in cell wall remodelling. Commun. Biol..

[16] Herkert P.F., Amatuzzi R.F., Alves L.R., Rodrigues M.L. (2019). Extracellular Vesicles as Vehicles for the Delivery of Biologically Active Fungal Molecules. Curr. Protein Pept. Sci..

[17] Alves L.R., Peres da Silva R., Sanchez D.A., Zamith-Miranda D., Rodrigues M.L., Goldenberg S., Puccia R., Nosanchuk J.D. (2019). Extracellular Vesicle-Mediated RNA Release in *Histoplasma capsulatum*. mSphere.

[18] Rodrigues M.L., Franzen A.J., Nimrichter L., Miranda K. (2013). Vesicular mechanisms of traffic of fungal molecules to the extracellular space. Curr. Opin. Microbiol..

[19] Matos Baltazar L., Nakayasu E.S., Sobreira T.J., Choi H., Casadevall A., Nimrichter L., Nosanchuk J.D. (2016). Antibody binding alters the characteristics and contents of extracellular vesicles released by *Histoplasma capsulatum*. mSphere.

[20] Baltazar L.M., Zamith-Miranda D., Burnet M.C., Choi H., Nimrichter L., Nakayasu E.S., Nosanchuk J.D. (2018). Concentration-dependent protein loading of extracellular vesicles released by *Histoplasma capsulatum* after antibody treatment and its modulatory action upon macrophages. Sci. Rep..

[21] de Toledo Martins S., Szwarc P., Goldenberg S., Alves L.R. (2019). Extracellular Vesicles in Fungi: Composition and Functions. Curr. Top. Microbiol. Immunol..

[22] Albuquerque P.C., Nakayasu E.S., Rodrigues M.L., Frases S., Casadevall A., Zancope-Oliveira R.M., Almeida I.C., Nosanchuk J.D. (2008). Vesicular transport in *Histoplasma capsulatum*: An effective mechanism for trans-cell wall transfer of proteins and lipids in ascomycetes. Cell Microbiol..

[23] Rodrigues M.L., Nakayasu E.S., Oliveira D.L., Nimrichter L., Nosanchuk J.D., Almeida I.C., Casadevall A. (2008). Extracellular vesicles produced by *Cryptococcus neoformans* contain protein components associated with virulence. Eukaryot Cell.

[24] Vallejo M.C., Matsuo A.L., Ganiko L., Medeiros L.C., Miranda K., Silva L.S., Freymüller-Haapalainen E., Sinigaglia-Coimbra R., Almeida I.C., Puccia R. (2011). The pathogenic fungus *Paracoccidioides brasiliensis* exports extracellular vesicles containing highly immunogenic α-Galactosyl epitopes. Eukaryot Cell.

[25] Silva B.M., Prados-Rosales R., Espadas-Moreno J., Wolf J.M., Luque-Garcia J.L., Gonçalves T., Casadevall A. (2014). Characterization of *Alternaria infectoria* extracellular vesicles. Med. Mycol..

[26] Gil-Bona A., Llama-Palacios A., Parra C.M., Vivanco F., Nombela C., Monteoliva L., Gil C. (2015). Proteomics unravels extracellular vesicles as carriers of classical cytoplasmic proteins in *Candida albicans*. J. Proteome Res..

[27] Vargas G., Rocha J.D., Oliveira D.L., Albuquerque P.C., Frases S., Santos S.S., Nosanchuk J.D., Gomes A.M., Medeiros L.C., Miranda K. (2015). Compositional and immunobiological analyses of extracellular vesicles released by *Candida albicans*. Cell Microbiol..

[28] Johansson H.J., Vallhov H., Holm T., Gehrmann U., Andersson A., Johansson C., Blom H., Carroni M., Lehtiö J., Scheynius A. (2018). Extracellular nanovesicles released from the commensal yeast *Malassezia sympodialis* are enriched in allergens and interact with cells in human skin. Sci. Rep..

[29] Zamith-Miranda D., Nimrichter L., Rodrigues M.L., Nosanchuk J.D. (2018). Fungal extracellular vesicles: Modulating host-pathogen interactions by both the fungus and the host. Microbes Infect..

[30] Konečná K., Klimentová J., Benada O., Němečková I., Janďourek O., Jílek P., Vejsová M. (2019). A comparative analysis of protein virulence factors released via extracellular vesicles in two *Candida albicans* strains cultivated in a nutrient-limited medium. Microb. Pathog..

[31] Oliveira D.L., Freire-de-Lima C.G., Nosanchuk J.D., Casadevall A., Rodrigues M.L., Nimrichter L. (2010). Extracellular vesicles from *Cryptococcus neoformans* modulate macrophage functions. Infect. Immun..

[32] Gehrmann U., Qazi K.R., Johansson C., Hultenby K., Karlsson M., Lundeberg L., Gabrielsson S., Scheynius A. (2011). Nanovesicles from *Malassezia sympodialis* and host exosomes induce cytokine responses – novel mechanisms for host-microbe interactions in atopic eczema. PLoS ONE.

[33] Da Silva T.A., Roque-Barreira M.C., Casadevall A., Almeida F. (2016). Extracellular vesicles from *Paracoccidioides brasiliensis* induced M1 polarization in vitro. Sci. Rep..

[34] Wolf J.M., Espadas J., Luque-Garcia J., Reynolds T., Casadevall A. (2015). Lipid biosynthetic genes affect *Candida albicans* extracellular vesicle morphology, cargo, and immunostimulatory properties. Eukaryot Cell.

[35] Benson J.R., Hare P.E. (1975). O-phthalaldehyde: Fluorogenic detection of primary amines in the picomole range. Comparison with fluorescamine and ninhydrin. Proc. Natl. Acad. Sci. USA.

[36] Church F.C., Swaisgood H.E., Porter D.H., Catignani G.L. (1983). Spectrophotometric assay using o-phthaldialdehyde for determination of proteolysis in milk and isolated milk proteins. J. Dair. Sci..

[37] Karkowska-Kuleta J., Satala D., Bochenska O., Rapala-Kozik M., Kozik A. (2019). Moonlighting proteins are variably exposed at the cell surfaces of *Candida glabrata, Candida parapsilosis* and *Candida tropicalis* under certain growth conditions. BMC Microbiol..

[38] Karkowska-Kuleta J., Zajac D., Bochenska O., Kozik A. (2015). Surfaceome of pathogenic yeasts, *Candida parapsilosis* and *Candida tropicalis*, revealed with the use of cell surface shaving method and shotgun proteomic approach. Acta Biochim. Pol..

[39] Karkowska-Kuleta J., Zajac D., Bras G., Bochenska O., Rapala-Kozik M., Kozik A. (2017). Binding of human plasminogen and high-molecular-mass kininogen by cell surface-exposed proteins of *Candida parapsilosis*. Acta Biochim. Pol..

[40] Théry C., Witwer K.W., Aikawa E., Alcaraz M.J., Anderson J.D., Andriantsitohaina R., Antoniou A., Arab T., Archer F., Atkin-Smith G.K. (2018). Minimal information for studies of extracellular vesicles 2018 (MISEV2018): A position statement of the International Society for Extracellular Vesicles and update of the MISEV2014 guidelines. J. Extracell. Vesicles.

[41] Hildonen S., Skarpen E., Halvorsen T.G., Reubsaet L. (2016). Isolation and mass spectrometry analysis of urinary extraexosomal proteins. Sci. Rep..

[42] Saari H., Lázaro-Ibáñez E., Viitala T., Vuorimaa-Laukkanen E., Siljander P., Yliperttula M. (2015). Microvesicle- and exosome-mediated drug delivery enhances the cytotoxicity of Paclitaxel in autologous prostate cancer cells. J. Control. Release.

[43] Arnaud M.B., Costanzo M.C., Skrzypek M.S., Binkley G., Lane C., Miyasato S.R., Sherlock G. (2005). The *Candida* genome database (CGD), a community resource for *Candida albicans* gene and protein information. Nucleic Acids Res..

[44] Cherry J.M., Hong E.L., Amundsen C., Balakrishnan R., Binkley G., Chan E.T., Christie K.R., Costanzo M.C., Dwight S.S., Engel S.R. (2012). *Saccharomyces* genome database: The genomics resource of budding yeast. Nucleic Acids Res..

[45] (2019). The UniProt Consortium. UniProt: A worldwide hub of protein knowledge. Nucleic Acids Res..

[46] Woith E., Fuhrmann G., Melzig M.F. (2019). Extracellular Vesicles-Connecting Kingdoms. Int. J. Mol. Sci..

[47] Bielska E., May R.C. (2019). Extracellular vesicles of human pathogenic fungi. Curr. Opin. Microbiol..

[48] Zarnowski R., Sanchez H., Covelli A.S., Dominguez E., Jaromin A., Bernhardt J., Mitchell K.F., Heiss C., Azadi P., Mitchell A. (2018). *Candida albicans* biofilm–induced vesicles confer drug resistance through matrix biogenesis. PLoS Biol..

[49] Nimrichter L., de Souza M.M., Del Poeta M., Nosanchuk J.D., Joffe L., Tavares P., Rodrigues M.L. (2016). Extracellular Vesicle-Associated Transitory Cell Wall Components and Their Impact on the Interaction of Fungi with Host Cells. Front. Microbiol..

[50] Hall R.A., Gow N.A. (2013). Mannosylation in *Candida albicans*: Role in cell wall function and immune recognition. Mol. Microbiol..

[51] Granger B.L., Flenniken M.L., Davis D.A., Mitchell A.P., Cutler J.E. (2005). Yeast wall protein 1 of *Candida albicans*. Microbiology.

[52] Granger B.L. (2018). Accessibility and contribution to glucan masking of natural and genetically tagged versions of yeast wall protein 1 of *Candida albicans*. PLoS ONE.

[53] Pietrella D., Bistoni G., Corbucci C., Perito S., Vecchiarelli A. (2006). *Candida albicans* mannoprotein influences the biological function of dendritic cells. Cell Microbiol..

[54] Sandini S., La Valle R., De Bernardis F., Macrì C., Cassone A. (2007). The 65 kDa mannoprotein gene of *Candida albicans* encodes a putative beta-glucanase adhesin required for hyphal morphogenesis and experimental pathogenicity. Cell Microbiol..

[55] MacCallum D.M., Castillo L., Nather K., Munro C.A., Brown A.J., Gow N.A., Odds F.C. (2009). Property differences among the four major *Candida albicans* strain clades. Eukaryot. Cell.

[56] Zaugg C., Borg-Von Zepelin M., Reichard U., Sanglard D., Monod M. (2001). Secreted aspartic proteinase family of *Candida tropicalis*. Infect. Immun..

[57] Kaur R., Ma B., Cormack B.P. (2007). A family of glycosylphosphatidylinositol-linked aspartyl proteases is required for virulence of *Candida glabrata*. Proc. Natl. Acad. Sci. USA.

[58] Yu S., Li W., Liu X., Che J., Wu Y., Lu J. (2016). Distinct Expression Levels of ALS, LIP, and SAP Genes in *Candida tropicalis* with Diverse Virulent Activities. Front. Microbiol..

[59] Sarthy A.V., McGonigal T., Coen M., Frost D.J., Meulbroek J.A., Goldman R.C. (1997). Phenotype in *Candida albicans* of a disruption of the BGL2 gene encoding a 1,3-beta-glucosyltransferase. Microbiology.

[60] De Bernardis F., Mühlschlegel F.A., Cassone A., Fonzi W.A. (1998). The pH of the host niche controls gene expression in and virulence of *Candida albicans*. Infect. Immun.

[61] Miura N., Ueda M. (2018). Evaluation of Unconventional Protein Secretion by *Saccharomyces cerevisiae* and other Fungi. Cells.

[62] Gil-Bona A., Amador-García A., Gil C., Monteoliva L. (2018). The external face of *Candida albicans*: A proteomic view of the cell surface and the extracellular environment. J. Proteom..

[63] Jeffery C.J. (1999). Moonlighting proteins. Trends Biochem. Sci..

[64] Karkowska-Kuleta J., Kozik A. (2014). Moonlighting proteins as virulence factors of pathogenic fungi, parasitic protozoa and multicellular parasites. Mol. Oral Microbiol..

[65] Gancedo C., Flores C.L., Gancedo J.M. (2016). The Expanding Landscape of Moonlighting Proteins in Yeasts. Microbiol Mol Biol Rev..

[66] Kozik A., Karkowska-Kuleta J., Zajac D., Bochenska O., Kedracka-Krok S., Jankowska U., Rapala-Kozik M. (2015). Fibronectin-, vitronectin- and laminin-binding proteins at the cell walls of *Candida parapsilosis* and *Candida tropicalis* pathogenic yeasts. BMC Microbiol..

[67] Karkowska-Kuleta J., Zajac D., Bras G., Bochenska O., Seweryn K., Kedracka-Krok S., Jankowska U., Rapala-Kozik M., & Kozik A. (2016). Characterization of the interactions between human high-molecular-mass kininogen and cell wall proteins of pathogenic yeasts *Candida tropicalis*. Acta Biochim. Pol..

[68] Swoboda R.K., Bertram G., Hollander H., Greenspan D., Greenspan J.S., Gow N.A., Gooday G.W., Brown A.J. (1993). Glycolytic enzymes of *Candida albicans* are nonubiquitous immunogens during candidiasis. Infect. Immun..

[69] Klotz S.A., Pendrak M.L., Hein R.C. (2001). Antibodies to alpha5beta1 and alpha(v)beta3 integrins react with *Candida albicans* alcohol dehydrogenase. Microbiology.

[70] Liu Y., Ou Y., Sun L., Li W., Yang J., Zhang X., Hu Y. (2019). Alcohol dehydrogenase of *Candida albicans* triggers differentiation of THP-1 cells into macrophages. J. Adv. Res..

[71] Lee P.Y., Gam L.H., Yong V.C., Rosli R., Ng K.P., Chong P.P. (2014). Identification of immunogenic proteins of *Candida parapsilosis* by serological proteome analysis. J. Appl. Microbiol..

[72] Lee P.Y., Gam L.H., Yong V.C., Rosli R., Ng K.P., Chong P.P. (2014). Immunoproteomic analysis of antibody response to cell wall-associated proteins of *Candida tropicalis*. J. Appl. Microbiol..

[73] Oliveira D.L., Nakayasu E.S., Joffe L.S., Guimaraes A.J., Sobreira T.J., Nosanchuk J.D., Cordero R.J., Frases S., Casadevall A., Almeida I.C. (2010). Characterization of yeast extracellular vesicles, evidence for the participation of different pathways of cellular traffic in vesicle biogenesis. PLoS ONE.

