# Supplementary material for: Characteristics of Extracellular Vesicles Released by the Pathogenic Yeast-Like Fungi Candida glabrata, Candida parapsilosis and Candida tropicalis
Source: Cells. 2020 Jul 18;9(7):1722. doi: 10.3390/cells9071722 (PMC7408413; doi:10.3390/cells9071722)
Supplement: Supplementary file 1 [file cells-09-01722-s001.zip › Supplementary table 3.pdf]

Supplementary Table 3. Mass spectrometry identification of *C. tropicalis* proteins in EVs after vesicle surface shaving with trypsin, vesicle sonication, or the preparation of fractions enriched with membrane proteins.

The resulting peptides were analyzed using the Dionex Ultimate 3000 UHPLC system coupled to an HCTUltra ETDII mass spectrometer and the obtained lists of peaks were searched against the NCBI protein database using an in-house Mascot server.

Accession number	Protein	Molecular mass [Da]	Score	Matches	Sequences	Sequence coverage [%]
**surface shaving with trypsin 1**						
gi|255728723	opaque-phase-specific protein OP4 precursor [*Candida tropicalis* MYA-3404]	43376	655	16	8	26
gi|255733002	plasma membrane ATPase 1 [*Candida tropicalis* MYA-3404]	98358	570	10	10	11
gi|255729274	conserved hypothetical protein [*Candida tropicalis* MYA-3404]	37686	386	10	5	19
gi|255729942	hypothetical protein CTRG_04193 [*Candida tropicalis* MYA-3404]	120876	384	9	8	8
gi|255731223	conserved hypothetical protein [*Candida tropicalis* MYA-3404]	38035	376	8	6	22
gi|255722347	hypothetical protein CTRG_00890 [*Candida tropicalis* MYA-3404]	53021	323	6	6	15
gi|255729820	conserved hypothetical protein [*Candida tropicalis* MYA-3404]	35427	216	5	4	13
gi|255729832	conserved hypothetical protein [*Candida tropicalis* MYA-3404]	21663	216	5	4	22
gi|255730149	protein EPD1 precursor [*Candida tropicalis* MYA-3404]	59740	211	4	4	9
gi|255727428	phosphoglycerate kinase [*Candida tropicalis* MYA-3404]	44686	166	5	5	14
gi|255725506	conserved hypothetical protein [*Candida tropicalis* MYA-3404]	31403	138	3	2	12
gi|255720907	glucan 1,3-beta-glucosidase precursor [*Candida tropicalis* MYA-3404]	34181	129	3	3	12

gi|255732890	glyceraldehyde-3-phosphate dehydrogenase [*Candida tropicalis* MYA-3404]	36209	127	3	3	11
gi|255722852	predicted protein [*Candida tropicalis* MYA-3404]	194842	119	3	3	1
gi|255732910	predicted protein [*Candida tropicalis* MYA-3404]	77134	112	3	3	3
gi|255721523	predicted protein [*Candida tropicalis* MYA-3404]	98965	106	2	2	3
gi|255729440	protein EPD2 precursor [*Candida tropicalis* MYA-3404]	61273	102	2	2	4
gi|255732521	alcohol dehydrogenase 2 [*Candida tropicalis* MYA-3404]	37181	99	3	3	9
gi|255723898	conserved hypothetical protein [*Candida tropicalis* MYA-3404]	65882	98	1	1	2
gi|255725714	hypothetical protein CTRG_02093 [*Candida tropicalis* MYA-3404]	39082	90	3	2	8
gi|255727360	conserved hypothetical protein [*Candida tropicalis* MYA-3404]	18653	82	4	4	35
gi|255722954	lysophospholipase 1 precursor [*Candida tropicalis* MYA-3404]	70009	80	2	2	3
gi|220900339	secreted aspartyl protease [*Candida tropicalis*]	31024	71	1	1	3
gi|255727881	enolase 1 [*Candida tropicalis* MYA-3404]	46984	70	2	2	7
gi|255731107	predicted protein [*Candida tropicalis* MYA-3404]	48865	68	2	2	5
gi|255728237	predicted protein [*Candida tropicalis* MYA-3404]	22931	58	1	1	5
gi|255725194	elongation factor 1-alpha [*Candida tropicalis* MYA-3404]	50336	49	2	2	3
**surface shaving with trypsin 2**						
gi|255728723	opaque-phase-specific protein OP4 precursor [*Candida tropicalis* MYA-3404]	43376	656	24	9	29
gi|255727428	phosphoglycerate kinase [*Candida tropicalis* MYA-3404]	44686	418	9	8	24
gi|255729274	conserved hypothetical protein [*Candida tropicalis* MYA-3404]	37686	406	9	5	19

gi|255729942	hypothetical protein CTRG_04193 [*Candida tropicalis* MYA-3404]	120876	397	8	8	9
gi|255722347	hypothetical protein CTRG_00890 [*Candida tropicalis* MYA-3404]	53021	349	6	5	15
gi|255731223	conserved hypothetical protein [*Candida tropicalis* MYA-3404]	38035	327	7	5	16
gi|255729820	conserved hypothetical protein [*Candida tropicalis* MYA-3404]	35427	290	6	4	16
gi|255729832	conserved hypothetical protein [*Candida tropicalis* MYA-3404]	21663	290	6	4	27
gi|255723898	conserved hypothetical protein [*Candida tropicalis* MYA-3404]	65882	285	4	4	8
gi|255723403	alcohol dehydrogenase 1 [*Candida tropicalis* MYA-3404]	43927	264	6	6	17
gi|255732890	glyceraldehyde-3-phosphate dehydrogenase [*Candida tropicalis* MYA-3404]	36209	251	5	4	14
gi|255733002	plasma membrane ATPase 1 [*Candida tropicalis* MYA-3404]	98358	223	4	4	5
gi|255721523	predicted protein [*Candida tropicalis* MYA-3404]	98965	202	3	3	4
gi|255725930	predicted protein [*Candida tropicalis* MYA-3404]	71216	194	7	6	8
gi|255720907	glucan 1,3-beta-glucosidase precursor [*Candida tropicalis* MYA-3404]	34181	184	5	4	12
gi|255725506	conserved hypothetical protein [*Candida tropicalis* MYA-3404]	31403	156	4	2	12
gi|255730873	predicted protein [*Candida tropicalis* MYA-3404]	23247	118	3	2	15
gi|255722852	predicted protein [*Candida tropicalis* MYA-3404]	194842	110	3	3	1
gi|255732910	predicted protein [*Candida tropicalis* MYA-3404]	77134	101	3	3	3
gi|255730149	protein EPD1 precursor [*Candida tropicalis* MYA-3404]	59740	87	2	2	5
gi|255729208	pyruvate decarboxylase [*Candida tropicalis* MYA-3404]	62500	79	2	2	7
gi|255728149	glucoamylase 1 precursor [*Candida tropicalis* MYA-3404]	102877	78	2	2	3
gi|255721095	hypothetical protein CTRG_00263 [*Candida tropicalis* MYA-3404]	49693	77	1	1	3

gi|255722954	lysophospholipase 1 precursor [*Candida tropicalis* MYA-3404]	70009	74	3	2	2
gi|255724450	pyruvate kinase [*Candida tropicalis* MYA-3404]	55729	65	2	2	6
gi|220900339	secreted aspartyl protease [*Candida tropicalis*]	31024	64	2	2	7
gi|255724160	elongation factor 2 [*Candida tropicalis* MYA-3404]	92277	59	2	2	2
gi|255722876	predicted protein [*Candida tropicalis* MYA-3404]	15870	58	1	1	5
gi|255728237	predicted protein [*Candida tropicalis* MYA-3404]	22931	54	1	1	5
**surface shaving with trypsin 3**						
gi|255728723	opaque-phase-specific protein OP4 precursor [*Candida tropicalis* MYA-3404]	43376	570	15	8	23
gi|255731223	conserved hypothetical protein [*Candida tropicalis* MYA-3404]	38035	424	8	6	22
gi|255733002	plasma membrane ATPase 1 [*Candida tropicalis* MYA-3404]	98358	371	6	6	8
gi|255722347	hypothetical protein CTRG_00890 [*Candida tropicalis* MYA-3404]	53021	353	7	6	15
gi|255729942	hypothetical protein CTRG_04193 [*Candida tropicalis* MYA-3404]	120876	313	8	8	8
gi|255727428	phosphoglycerate kinase [*Candida tropicalis* MYA-3404]	44686	309	8	8	22
gi|255729274	conserved hypothetical protein [*Candida tropicalis* MYA-3404]	37686	291	6	4	13
gi|255732890	glyceraldehyde-3-phosphate dehydrogenase [*Candida tropicalis* MYA-3404]	36209	234	6	6	21
gi|255723898	conserved hypothetical protein [*Candida tropicalis* MYA-3404]	65882	220	3	3	6
gi|255725506	conserved hypothetical protein [*Candida tropicalis* MYA-3404]	31403	210	3	3	14
gi|255723403	alcohol dehydrogenase 1 [*Candida tropicalis* MYA-3404]	43927	177	5	4	13
gi|255729820	conserved hypothetical protein [*Candida tropicalis* MYA-3404]	35427	160	4	3	10

gi|255729832	conserved hypothetical protein [*Candida tropicalis* MYA-3404]	21663	160	4	3	17
gi|255732521	alcohol dehydrogenase 2 [*Candida tropicalis* MYA-3404]	37181	150	5	4	14
gi|255721523	predicted protein [*Candida tropicalis* MYA-3404]	98965	149	2	2	3
gi|255722852	predicted protein [*Candida tropicalis* MYA-3404]	194842	111	3	3	1
gi|255724450	pyruvate kinase [*Candida tropicalis* MYA-3404]	55729	106	2	2	5
gi|255729440	protein EPD2 precursor [*Candida tropicalis* MYA-3404]	61273	102	2	2	4
gi|255730149	protein EPD1 precursor [*Candida tropicalis* MYA-3404]	59740	99	2	2	5
gi|255732093	ADP,ATP carrier protein [*Candida tropicalis* MYA-3404]	33532	86	2	2	7
gi|255722021	transaldolase [*Candida tropicalis* MYA-3404]	35641	78	2	2	5
gi|255722954	lysophospholipase 1 precursor [*Candida tropicalis* MYA-3404]	70009	76	2	2	4
gi|255727360	conserved hypothetical protein [*Candida tropicalis* MYA-3404]	18653	74	3	3	26
gi|255720907	glucan 1,3-beta-glucosidase precursor [*Candida tropicalis* MYA-3404]	34181	71	1	1	3
gi|255728149	glucoamylase 1 precursor [*Candida tropicalis* MYA-3404]	102877	70	2	2	2
gi|255731268	hypothetical protein CTRG_04856 [*Candida tropicalis* MYA-3404]	34741	69	2	2	9
gi|255730873	predicted protein [*Candida tropicalis* MYA-3404]	23247	63	1	1	8
gi|255731592	peptidyl-prolyl cis-trans isomerase [*Candida tropicalis* MYA-3404]	14363	56	2	2	12
gi|220900339	secreted aspartyl protease [*Candida tropicalis*]	31024	53	1	1	3
**surface shaving with trypsin 4**						
gi|255720907	glucan 1,3-beta-glucosidase precursor [*Candida tropicalis* MYA-3404]	34181	432	11	8	28
gi|255729820	conserved hypothetical protein [*Candida tropicalis* MYA-3404]	35427	305	7	5	19

gi|255729832	conserved hypothetical protein [*Candida tropicalis* MYA-3404]	21663	305	7	5	31
gi|255722347	hypothetical protein CTRG_00890 [*Candida tropicalis* MYA-3404]	53021	294	6	6	15
gi|255725714	hypothetical protein CTRG_02093 [*Candida tropicalis* MYA-3404]	39082	182	5	4	17
gi|220900339	secreted aspartyl protease [*Candida tropicalis*]	31024	164	5	4	10
gi|255730873	predicted protein [*Candida tropicalis* MYA-3404]	23247	149	4	3	26
gi|255728571	predicted protein [*Candida tropicalis* MYA-3404]	49064	121	3	3	6
gi|255727428	phosphoglycerate kinase [*Candida tropicalis* MYA-3404]	44686	96	3	3	8
gi|255722954	lysophospholipase 1 precursor [*Candida tropicalis* MYA-3404]	70009	93	2	2	3
gi|255731592	peptidyl-prolyl cis-trans isomerase [*Candida tropicalis* MYA-3404]	14363	85	3	3	22
gi|255729942	hypothetical protein CTRG_04193 [*Candida tropicalis* MYA-3404]	120876	69	2	2	2
gi|255727881	enolase 1 [*Candida tropicalis* MYA-3404]	46984	65	2	2	7
gi|255732890	glyceraldehyde-3-phosphate dehydrogenase [*Candida tropicalis* MYA-3404]	36209	61	2	2	6
gi|255724074	predicted protein [*Candida tropicalis* MYA-3404]	163634	54	1	1	0
**vesicles sonication 1**						
gi|255727428	phosphoglycerate kinase [*Candida tropicalis* MYA-3404]	44686	856	22	19	52
gi|255729942	hypothetical protein CTRG_04193 [*Candida tropicalis* MYA-3404]	120876	805	18	17	17
gi|255728723	opaque-phase-specific protein OP4 precursor [*Candida tropicalis* MYA-3404]	43376	567	12	10	26
gi|255728149	glucoamylase 1 precursor [*Candida tropicalis* MYA-3404]	102877	455	12	11	11

gi|255725714	hypothetical protein CTRG_02093 [*Candida tropicalis* MYA-3404]	39082	404	10	9	35
gi|255724450	pyruvate kinase [*Candida tropicalis* MYA-3404]	55729	401	9	9	20
gi|255720907	glucan 1,3-beta-glucosidase precursor [*Candida tropicalis* MYA-3404]	34181	379	10	9	27
gi|255729274	conserved hypothetical protein [*Candida tropicalis* MYA-3404]	37686	308	6	4	15
gi|255725930	predicted protein [*Candida tropicalis* MYA-3404]	71216	291	7	7	12
gi|255727881	enolase 1 [*Candida tropicalis* MYA-3404]	46984	274	6	6	21
gi|255731223	conserved hypothetical protein [*Candida tropicalis* MYA-3404]	38035	265	4	4	12
gi|255721523	predicted protein [*Candida tropicalis* MYA-3404]	98965	249	6	6	6
gi|255731268	hypothetical protein CTRG_04856 [*Candida tropicalis* MYA-3404]	34741	236	6	6	21
gi|255733002	plasma membrane ATPase 1 [*Candida tropicalis* MYA-3404]	98358	227	5	5	6
gi|255729820	conserved hypothetical protein [*Candida tropicalis* MYA-3404]	35427	210	5	4	13
gi|255729832	conserved hypothetical protein [*Candida tropicalis* MYA-3404]	21663	210	5	4	22
gi|255723403	alcohol dehydrogenase 1 [*Candida tropicalis* MYA-3404]	43927	201	6	6	17
gi|255730427	eukaryotic initiation factor 4A [*Candida tropicalis* MYA-3404]	44795	176	4	4	10
gi|255722852	predicted protein [*Candida tropicalis* MYA-3404]	194842	171	8	4	3
gi|261412043	alcohol dehydrogenase [*Candida tropicalis*]	33332	168	5	5	19
gi|255730873	predicted protein [*Candida tropicalis* MYA-3404]	23247	161	3	2	15
gi|255720595	ornithine aminotransferase [*Candida tropicalis* MYA-3404]	47518	155	3	3	7
gi|255732521	alcohol dehydrogenase 2 [*Candida tropicalis* MYA-3404]	37181	154	5	5	18
gi|255731107	predicted protein [*Candida tropicalis* MYA-3404]	48865	151	6	5	13
gi|255732780	conserved hypothetical protein [*Candida tropicalis* MYA-3404]	50823	127	5	4	12

gi|255729208	pyruvate decarboxylase [*Candida tropicalis* MYA-3404]	62500	119	2	2	4
gi|255725820	glycolipid 2-alpha-mannosyltransferase [*Candida tropicalis* MYA-3404]	50244	119	2	2	4
gi|255725506	conserved hypothetical protein [*Candida tropicalis* MYA-3404]	31403	109	3	3	15
gi|255728237	predicted protein [*Candida tropicalis* MYA-3404]	22931	108	2	2	10
gi|255724074	predicted protein [*Candida tropicalis* MYA-3404]	163634	106	2	2	1
gi|255730273	hypothetical protein CTRG_04358 [*Candida tropicalis* MYA-3404]	84100	99	2	2	2
gi|255729062	pH-regulated antigen PRA1 precursor [*Candida tropicalis* MYA-3404]	35235	88	3	2	7
gi|255723898	conserved hypothetical protein [*Candida tropicalis* MYA-3404]	65882	85	1	1	2
gi|255721021	malate dehydrogenase, mitochondrial precursor [*Candida tropicalis* MYA-3404]	34822	81	3	3	11
gi|255725240	predicted protein [*Candida tropicalis* MYA-3404]	66682	77	3	3	6
gi|255730537	candidapepsin-7 precursor [*Candida tropicalis* MYA-3404]	73220	73	2	2	3
gi|255725372	predicted protein [*Candida tropicalis* MYA-3404]	76231	68	1	1	1
gi|255725374	conserved hypothetical protein [*Candida tropicalis* MYA-3404]	165466	68	1	1	0
gi|255725376	conserved hypothetical protein [*Candida tropicalis* MYA-3404]	83759	68	1	1	1
gi|255732093	ADP,ATP carrier protein [*Candida tropicalis* MYA-3404]	33532	57	2	2	7
**vesicles sonication 2**						
gi|255728723	opaque-phase-specific protein OP4 precursor [*Candida tropicalis* MYA-3404]	43376	612	13	8	24
gi|255733002	plasma membrane ATPase 1 [*Candida tropicalis* MYA-3404]	98358	554	15	14	17

gi|255729942	hypothetical protein CTRG_04193 [*Candida tropicalis* MYA-3404]	120876	379	12	11	13
gi|255720907	glucan 1,3-beta-glucosidase precursor [*Candida tropicalis* MYA-3404]	34181	375	9	9	25
gi|255722347	hypothetical protein CTRG_00890 [*Candida tropicalis* MYA-3404]	53021	297	6	6	15
gi|255731223	conserved hypothetical protein [*Candida tropicalis* MYA-3404]	38035	287	5	5	13
gi|255729274	conserved hypothetical protein [*Candida tropicalis* MYA-3404]	37686	260	6	4	15
gi|255727428	phosphoglycerate kinase [*Candida tropicalis* MYA-3404]	44686	237	9	9	27
gi|255731057	conserved hypothetical protein [*Candida tropicalis* MYA-3404]	29766	228	6	6	24
gi|255722852	predicted protein [*Candida tropicalis* MYA-3404]	194842	162	6	5	4
gi|255732780	conserved hypothetical protein [*Candida tropicalis* MYA-3404]	50823	162	4	4	12
gi|255730873	predicted protein [*Candida tropicalis* MYA-3404]	23247	157	4	3	25
gi|255721523	predicted protein [*Candida tropicalis* MYA-3404]	98965	154	3	3	3
gi|255732093	ADP,ATP carrier protein [*Candida tropicalis* MYA-3404]	33532	150	5	4	15
gi|255725714	hypothetical protein CTRG_02093 [*Candida tropicalis* MYA-3404]	39082	148	3	3	9
gi|255729820	conserved hypothetical protein [*Candida tropicalis* MYA-3404]	35427	142	3	3	13
gi|255729832	conserved hypothetical protein [*Candida tropicalis* MYA-3404]	21663	142	3	3	22
gi|255732521	alcohol dehydrogenase 2 [*Candida tropicalis* MYA-3404]	37181	130	4	4	12
gi|255727881	enolase 1 [*Candida tropicalis* MYA-3404]	46984	128	4	4	13
gi|255725506	conserved hypothetical protein [*Candida tropicalis* MYA-3404]	31403	97	2	2	8
gi|255721095	hypothetical protein CTRG_00263 [*Candida tropicalis* MYA-3404]	49693	81	2	2	5

gi|255729440	protein EPD2 precursor [*Candida tropicalis* MYA-3404]	61273	77	3	3	7
gi|255730149	protein EPD1 precursor [*Candida tropicalis* MYA-3404]	59740	71	2	2	4
gi|255728237	predicted protein [*Candida tropicalis* MYA-3404]	22931	65	1	1	5
gi|255731107	predicted protein [*Candida tropicalis* MYA-3404]	48865	62	1	1	3
**vesicles sonication 3**						
gi|255725714	hypothetical protein CTRG_02093 [*Candida tropicalis* MYA-3404]	39082	415	12	9	35
gi|255728723	opaque-phase-specific protein OP4 precursor [*Candida tropicalis* MYA-3404]	43376	186	4	4	12
gi|255729274	conserved hypothetical protein [*Candida tropicalis* MYA-3404]	37686	160	3	3	9
gi|255722852	predicted protein [*Candida tropicalis* MYA-3404]	194842	122	3	3	1
gi|255729942	hypothetical protein CTRG_04193 [*Candida tropicalis* MYA-3404]	120876	101	4	4	4
gi|255720907	glucan 1,3-beta-glucosidase precursor [*Candida tropicalis* MYA-3404]	34181	90	3	3	12
gi|255732910	predicted protein [*Candida tropicalis* MYA-3404]	77134	87	3	3	3
gi|255723898	conserved hypothetical protein [*Candida tropicalis* MYA-3404]	65882	78	3	2	4
gi|255729274	conserved hypothetical protein [*Candida tropicalis* MYA-3404]	37686	72	2	1	3
**fraction enriched with membrane proteins 1**						
gi|255728723	opaque-phase-specific protein OP4 precursor [*Candida tropicalis* MYA-3404]	43376	544	11	8	24
gi|255731223	conserved hypothetical protein [*Candida tropicalis* MYA-3404]	38035	252	4	3	10
gi|255729274	conserved hypothetical protein [*Candida tropicalis* MYA-3404]	37686	209	4	4	9

gi|255730873	predicted protein [*Candida tropicalis* MYA-3404]	23247	207	6	4	31
gi|255729942	hypothetical protein CTRG_04193 [*Candida tropicalis* MYA-3404]	120876	200	6	6	6
gi|255727428	phosphoglycerate kinase [*Candida tropicalis* MYA-3404]	44686	160	5	5	17
gi|255721523	predicted protein [*Candida tropicalis* MYA-3404]	98965	150	4	4	4
gi|255725930	predicted protein [*Candida tropicalis* MYA-3404]	71216	131	4	4	6
gi|255733002	plasma membrane ATPase 1 [*Candida tropicalis* MYA-3404]	98358	124	4	4	4
gi|255732521	alcohol dehydrogenase 2 [*Candida tropicalis* MYA-3404]	37181	115	4	4	18
gi|255722852	predicted protein [*Candida tropicalis* MYA-3404]	194842	113	3	3	1
gi|255725506	conserved hypothetical protein [*Candida tropicalis* MYA-3404]	31403	109	2	2	11
gi|255730149	protein EPD1 precursor [*Candida tropicalis* MYA-3404]	59740	75	2	2	4
**fraction enriched with membrane proteins 2**						
gi|255728723	opaque-phase-specific protein OP4 precursor [*Candida tropicalis* MYA-3404]	43376	391	12	7	18
gi|255729274	conserved hypothetical protein [*Candida tropicalis* MYA-3404]	37686	183	5	3	13
gi|255729820	conserved hypothetical protein [*Candida tropicalis* MYA-3404]	35427	165	3	3	13
gi|255729832	conserved hypothetical protein [*Candida tropicalis* MYA-3404]	21663	165	3	3	22
gi|255731223	conserved hypothetical protein [*Candida tropicalis* MYA-3404]	38035	115	3	3	9
gi|255725506	conserved hypothetical protein [*Candida tropicalis* MYA-3404]	31403	99	3	2	7
gi|255722852	predicted protein [*Candida tropicalis* MYA-3404]	194842	96	3	3	1
gi|255723032	predicted protein [*Candida tropicalis* MYA-3404]	15229	84	1	1	8
gi|255732780	conserved hypothetical protein [*Candida tropicalis* MYA-3404]	50823	84	1	1	2

gi|255732521	alcohol dehydrogenase 2 [*Candida tropicalis* MYA-3404]	37181	69	2	2	9
**fraction enriched with membrane proteins 3**						
gi|255728723	opaque-phase-specific protein OP4 precursor [*Candida tropicalis* MYA-3404]	43376	474	18	8	21
gi|255729274	conserved hypothetical protein [*Candida tropicalis* MYA-3404]	37686	246	8	4	13
gi|255731223	conserved hypothetical protein [*Candida tropicalis* MYA-3404]	38035	190	5	3	11
gi|255725930	predicted protein [*Candida tropicalis* MYA-3404]	71216	141	5	5	7
gi|255725506	conserved hypothetical protein [*Candida tropicalis* MYA-3404]	31403	124	3	2	12
gi|255722347	hypothetical protein CTRG_00890 [*Candida tropicalis* MYA-3404]	53021	94	2	2	5
gi|255722371	hypothetical protein CTRG_00902 [*Candida tropicalis* MYA-3404]	53033	94	2	2	5
gi|255732780	conserved hypothetical protein [*Candida tropicalis* MYA-3404]	50823	85	2	1	2
gi|255722852	predicted protein [*Candida tropicalis* MYA-3404]	194842	82	2	2	0
gi|255722876	predicted protein [*Candida tropicalis* MYA-3404]	15870	66	1	1	5
**fraction enriched with membrane proteins 4**						
gi|255728723	opaque-phase-specific protein OP4 precursor [*Candida tropicalis* MYA-3404]	43376	462	20	7	20
gi|255731223	conserved hypothetical protein [*Candida tropicalis* MYA-3404]	38035	436	9	7	26
gi|255729274	conserved hypothetical protein [*Candida tropicalis* MYA-3404]	37686	275	6	4	13
gi|255725506	conserved hypothetical protein [*Candida tropicalis* MYA-3404]	31403	102	3	2	12
gi|255722852	predicted protein [*Candida tropicalis* MYA-3404]	194842	79	2	2	0
gi|255730004	predicted protein [*Candida tropicalis* MYA-3404]	95636	79	2	2	1

gi|255732780	conserved hypothetical protein [*Candida tropicalis* MYA-3404]	50823	71	2	1	2
gi|255728237	predicted protein [*Candida tropicalis* MYA-3404]	22931	58	1	1	5
gi|255731029	conserved hypothetical protein [*Candida tropicalis* MYA-3404]	30545	54	2	2	5
