# Supplementary material for: Characteristics of Extracellular Vesicles Released by the Pathogenic Yeast-Like Fungi Candida glabrata, Candida parapsilosis and Candida tropicalis
Source: Cells. 2020 Jul 18;9(7):1722. doi: 10.3390/cells9071722 (PMC7408413; doi:10.3390/cells9071722)
Supplement: Supplementary file 1 [file cells-09-01722-s001.zip › Supplementary table 1.pdf]

Supplementary Table 1. Mass spectrometry identification of *C. glabrata* proteins in EVs after vesicle surface shaving with trypsin, vesicle sonication, or the preparation of fractions enriched with membrane proteins.

The resulting peptides were analyzed using the Dionex Ultimate 3000 UHPLC system coupled to an HCTUltra ETDII mass spectrometer and the obtained lists of peaks were searched against the NCBI protein database using an in-house Mascot server.

Accession number	Protein	Molecular mass [Da]	Score	Matches	Sequences	Sequence coverage [%]
**surface shaving with trypsin 1**						
gi|50292035	hypothetical protein [*Candida glabrata* CBS 138]	136648	825	27	21	20
gi|50294560	hypothetical protein [*Candida glabrata* CBS 138]	62152	595	14	12	28
gi|50289857	hypothetical protein [*Candida glabrata* CBS 138]	46767	595	12	10	28
gi|50293403	hypothetical protein [*Candida glabrata* CBS 138]	44704	590	13	13	35
gi|50284959	hypothetical protein [*Candida glabrata* CBS 138]	93783	572	9	9	13
gi|50288681	hypothetical protein [*Candida glabrata* CBS 138]	35985	563	13	10	33
gi|50286669	hypothetical protein [*Candida glabrata* CBS 138]	59098	546	17	9	23
gi|50289685	hypothetical protein [*Candida glabrata* CBS 138]	51468	475	14	13	29
gi|50294908	hypothetical protein [*Candida glabrata* CBS 138]	54975	465	13	11	26
gi|50295024	hypothetical protein [*Candida glabrata* CBS 138]	53878	402	7	7	20
gi|50287897	hypothetical protein [*Candida glabrata* CBS 138]	39767	367	7	7	27
gi|50292739	acetate-CoA ligase [*Candida glabrata* CBS 138]	79670	352	7	7	10
gi|50291073	hypothetical protein [*Candida glabrata* CBS 138]	37779	288	8	5	20

gi|50290317	hypothetical protein [*Candida glabrata* CBS 138]	37920	254	5	5	14
gi|50289283	hypothetical protein [*Candida glabrata* CBS 138]	42203	209	3	3	9
gi|50284865	hypothetical protein [*Candida glabrata* CBS 138]	62304	199	4	3	10
gi|50288687	hypothetical protein [*Candida glabrata* CBS 138]	49013	187	4	4	9
gi|50289307	hypothetical protein [*Candida glabrata* CBS 138]	60942	183	4	4	9
gi|50291585	hypothetical protein [*Candida glabrata* CBS 138]	72055	158	3	3	5
gi|50284869	hypothetical protein [*Candida glabrata* CBS 138]	30312	154	4	4	10
gi|50285099	hypothetical protein [*Candida glabrata* CBS 138]	54057	145	2	2	6
gi|9650673	actin [*Candida glabrata*]	36600	144	3	3	10
gi|18073449	GAS-1 homologue [*Candida glabrata*]	60675	141	3	3	4
gi|50287951	60S acidic ribosomal protein P0 [*Candida glabrata* CBS 138]	33572	133	3	3	14
gi|50287419	hypothetical protein [*Candida glabrata* CBS 138]	57350	131	2	2	5
gi|50286375	hypothetical protein [*Candida glabrata* CBS 138]	42980	95	3	2	9
gi|50294171	hypothetical protein [*Candida glabrata* CBS 138]	30205	87	2	2	11
gi|50286153	hypothetical protein [*Candida glabrata* CBS 138]	30297	84	2	2	6
gi|50290013	hypothetical protein [*Candida glabrata* CBS 138]	44930	81	2	2	4
gi|50285355	hypothetical protein [*Candida glabrata* CBS 138]	36811	75	1	1	4
gi|50292893	hypothetical protein [*Candida glabrata* CBS 138]	39503	72	3	3	6
gi|50285407	hypothetical protein [*Candida glabrata* CBS 138]	29899	72	3	3	13
gi|302309608	hypothetical protein [*Candida glabrata* CBS 138]	66558	69	2	2	3
gi|50294606	hypothetical protein [*Candida glabrata* CBS 138]	34283	66	2	2	8

gi|50289515	60S ribosomal protein L1 [*Candida glabrata* CBS 138]	24636	65	3	2	11
gi|50294608	hypothetical protein [*Candida glabrata* CBS 138]	22145	59	1	1	7
gi|50292597	hypothetical protein [*Candida glabrata* CBS 138]	20962	55	2	2	7
gi|50294025	hypothetical protein [*Candida glabrata* CBS 138]	43265	55	1	1	2
gi|50294400	hypothetical protein [*Candida glabrata* CBS 138]	77546	54	1	1	1
**surface shaving with trypsin 2**						
gi|50294908	hypothetical protein [*Candida glabrata* CBS 138]	54975	640	14	12	29
gi|50293403	hypothetical protein [*Candida glabrata* CBS 138]	44704	622	12	12	35
gi|50284959	hypothetical protein [*Candida glabrata* CBS 138]	93783	576	12	11	15
gi|50292035	hypothetical protein [*Candida glabrata* CBS 138]	136648	561	17	13	11
gi|50288681	hypothetical protein [*Candida glabrata* CBS 138]	35985	492	14	10	24
gi|50289857	hypothetical protein [*Candida glabrata* CBS 138]	46767	486	9	7	20
gi|50292739	acetate-CoA ligase [*Candida glabrata* CBS 138]	79670	472	11	9	13
gi|50289685	hypothetical protein [*Candida glabrata* CBS 138]	51468	420	12	11	29
gi|25992752	pyruvate decarboxylase [*Candida glabrata*]	62011	410	9	7	15
gi|50294560	hypothetical protein [*Candida glabrata* CBS 138]	62152	410	9	7	15
gi|50286669	hypothetical protein [*Candida glabrata* CBS 138]	59098	407	12	6	14
gi|50295024	hypothetical protein [*Candida glabrata* CBS 138]	53878	320	5	5	16
gi|50288687	hypothetical protein [*Candida glabrata* CBS 138]	49013	311	8	7	16
gi|50290013	hypothetical protein [*Candida glabrata* CBS 138]	44930	246	5	5	16
gi|50290317	hypothetical protein [*Candida glabrata* CBS 138]	37920	193	5	4	13

gi|50287737	hypothetical protein [*Candida glabrata* CBS 138]	21335	181	3	3	23
gi|50291073	hypothetical protein [*Candida glabrata* CBS 138]	37779	138	3	3	8
gi|50284869	hypothetical protein [*Candida glabrata* CBS 138]	30312	114	3	3	7
gi|50287897	hypothetical protein [*Candida glabrata* CBS 138]	39767	103	2	2	6
gi|50287073	hypothetical protein [*Candida glabrata* CBS 138]	27574	101	2	2	8
gi|50286085	hypothetical protein [*Candida glabrata* CBS 138]	74046	91	2	2	3
gi|50289307	hypothetical protein [*Candida glabrata* CBS 138]	60942	90	4	3	6
gi|50292893	hypothetical protein [*Candida glabrata* CBS 138]	39503	85	2	2	5
gi|50285099	hypothetical protein [*Candida glabrata* CBS 138]	54057	78	1	1	2
gi|50289283	hypothetical protein [*Candida glabrata* CBS 138]	42203	74	1	1	4
gi|50287951	60S acidic ribosomal protein P0 [*Candida glabrata* CBS 138]	33572	70	1	1	3
gi|50288777	40S ribosomal protein S5 [*Candida glabrata* CBS 138]	25194	69	1	1	6
gi|50285355	hypothetical protein [*Candida glabrata* CBS 138]	36811	64	1	1	3
gi|50289161	hypothetical protein [*Candida glabrata* CBS 138]	38094	59	2	2	8
gi|50292725	hypothetical protein [*Candida glabrata* CBS 138]	80983	59	1	1	1
gi|50287141	hypothetical protein [*Candida glabrata* CBS 138]	55434	55	1	1	2
**surface shaving with trypsin 3**						
gi|50284959	hypothetical protein [*Candida glabrata* CBS 138]	93783	611	13	13	17
gi|50294908	hypothetical protein [*Candida glabrata* CBS 138]	54975	568	14	11	29
gi|50288681	hypothetical protein [*Candida glabrata* CBS 138]	35985	535	15	12	38
gi|50292035	hypothetical protein [*Candida glabrata* CBS 138]	136648	511	20	12	10

gi|50286669	hypothetical protein [*Candida glabrata* CBS 138]	59098	480	14	8	18
gi|25992752	pyruvate decarboxylase [*Candida glabrata*]	62011	375	9	8	14
gi|50293403	hypothetical protein [*Candida glabrata* CBS 138]	44704	370	10	10	25
gi|50295024	hypothetical protein [*Candida glabrata* CBS 138]	53878	327	6	5	14
gi|50289857	hypothetical protein [*Candida glabrata* CBS 138]	46767	305	7	6	15
gi|50292739	acetate--CoA ligase [*Candida glabrata* CBS 138]	79670	296	6	6	8
gi|50289685	hypothetical protein [*Candida glabrata* CBS 138]	51468	293	7	6	14
gi|50285355	hypothetical protein [*Candida glabrata* CBS 138]	36811	229	5	5	16
gi|50287737	hypothetical protein [*Candida glabrata* CBS 138]	21335	227	3	3	23
gi|50288687	hypothetical protein [*Candida glabrata* CBS 138]	49013	212	7	6	14
gi|50287897	hypothetical protein [*Candida glabrata* CBS 138]	39767	184	3	3	10
gi|50290317	hypothetical protein [*Candida glabrata* CBS 138]	37920	181	5	5	15
gi|50284865	hypothetical protein [*Candida glabrata* CBS 138]	62304	168	3	3	7
gi|50290013	hypothetical protein [*Candida glabrata* CBS 138]	44930	165	4	4	12
gi|50292893	hypothetical protein [*Candida glabrata* CBS 138]	39503	147	3	3	7
gi|50289283	hypothetical protein [*Candida glabrata* CBS 138]	42203	142	2	2	7
gi|50284733	hypothetical protein [*Candida glabrata* CBS 138]	98941	120	2	2	2
gi|50292597	hypothetical protein [*Candida glabrata* CBS 138]	20962	117	4	4	26
gi|50287951	60S acidic ribosomal protein P0 [*Candida glabrata* CBS 138]	33572	109	3	2	6
gi|18073449	GAS-1 homologue [*Candida glabrata*]	60675	102	2	2	4
gi|50294205	60S ribosomal protein L25 [*Candida glabrata* CBS 138]	15695	101	2	2	18

gi|50284869	hypothetical protein [*Candida glabrata* CBS 138]	30312	99	3	3	8
gi|50289515	60S ribosomal protein L1 [*Candida glabrata* CBS 138]	24636	91	2	2	9
gi|50284867	hypothetical protein [*Candida glabrata* CBS 138]	61973	91	2	2	4
gi|50287007	hypothetical protein [*Candida glabrata* CBS 138]	66848	83	2	2	3
gi|50291211	hypothetical protein [*Candida glabrata* CBS 138]	57104	79	1	1	2
gi|50285099	hypothetical protein [*Candida glabrata* CBS 138]	54057	72	1	1	2
gi|50292725	hypothetical protein [*Candida glabrata* CBS 138]	80983	66	3	2	4
gi|50294171	hypothetical protein [*Candida glabrata* CBS 138]	30205	61	1	1	5
gi|50294025	hypothetical protein [*Candida glabrata* CBS 138]	43265	58	2	2	4
**surface shaving with trypsin 4**						
gi|50292035	hypothetical protein [*Candida glabrata* CBS 138]	136648	697	22	18	14
gi|50288681	hypothetical protein [*Candida glabrata* CBS 138]	35985	588	16	12	44
gi|50286669	hypothetical protein [*Candida glabrata* CBS 138]	59098	513	14	8	19
gi|50289857	hypothetical protein [*Candida glabrata* CBS 138]	46767	502	10	9	26
gi|50289685	hypothetical protein [*Candida glabrata* CBS 138]	51468	487	14	12	26
gi|50294560	hypothetical protein [*Candida glabrata* CBS 138]	62152	449	12	12	24
gi|50294908	hypothetical protein [*Candida glabrata* CBS 138]	54975	430	8	8	19
gi|50295024	hypothetical protein [*Candida glabrata* CBS 138]	53878	393	7	6	16
gi|50292739	acetate--CoA ligase [*Candida glabrata* CBS 138]	79670	330	11	11	16
gi|50287897	hypothetical protein [*Candida glabrata* CBS 138]	39767	310	5	4	14
gi|50291073	hypothetical protein [*Candida glabrata* CBS 138]	37779	227	5	4	13

gi|50293403	hypothetical protein [*Candida glabrata* CBS 138]	44704	224	5	5	14
gi|8927040	elongation factor 2 [*Candida glabrata*]	90394	221	6	6	10
gi|50290317	hypothetical protein [*Candida glabrata* CBS 138]	37920	153	3	3	8
gi|50284869	hypothetical protein [*Candida glabrata* CBS 138]	30312	135	3	3	8
gi|50290013	hypothetical protein [*Candida glabrata* CBS 138]	44930	116	3	3	8
gi|50292893	hypothetical protein [*Candida glabrata* CBS 138]	39503	114	4	3	10
gi|50286153	hypothetical protein [*Candida glabrata* CBS 138]	30297	81	2	2	5
gi|50294171	hypothetical protein [*Candida glabrata* CBS 138]	30205	78	2	1	5
gi|50292597	hypothetical protein [*Candida glabrata* CBS 138]	20962	76	3	3	12
gi|50285099	hypothetical protein [*Candida glabrata* CBS 138]	54057	75	1	1	2
gi|50287951	60S acidic ribosomal protein P0 [*Candida glabrata* CBS 138]	33572	73	2	2	6
gi|50289459	hypothetical protein [*Candida glabrata* CBS 138]	26968	62	1	1	6
gi|50293465	hypothetical protein [*Candida glabrata* CBS 138]	19705	61	1	1	7
**vesicles sonication 1**						
gi|50288681	hypothetical protein [*Candida glabrata* CBS 138]	35985	886	29	16	60
gi|50292035	hypothetical protein [*Candida glabrata* CBS 138]	136648	851	22	19	18
gi|452816300	acid trehalase [*Candida glabrata*]	136605	683	20	15	16
gi|50294908	hypothetical protein [*Candida glabrata* CBS 138]	54975	634	15	15	35
gi|50293403	hypothetical protein [*Candida glabrata* CBS 138]	44704	632	19	18	50
gi|50289857	hypothetical protein [*Candida glabrata* CBS 138]	46767	612	12	10	27
gi|50286669	hypothetical protein [*Candida glabrata* CBS 138]	59098	592	20	9	18

gi|50289685	hypothetical protein [*Candida glabrata* CBS 138]	51468	548	16	13	27
gi|25992752	pyruvate decarboxylase [*Candida glabrata*]	62011	532	17	13	26
gi|50294560	hypothetical protein [*Candida glabrata* CBS 138]	62152	532	17	13	26
gi|50284733	hypothetical protein [*Candida glabrata* CBS 138]	98941	502	8	8	10
gi|50288687	hypothetical protein [*Candida glabrata* CBS 138]	49013	429	11	10	23
gi|50287897	hypothetical protein [*Candida glabrata* CBS 138]	39767	388	8	7	27
gi|50290013	hypothetical protein [*Candida glabrata* CBS 138]	44930	357	9	8	21
gi|50290317	hypothetical protein [*Candida glabrata* CBS 138]	37920	355	9	8	26
gi|50287951	60S acidic ribosomal protein P0 [*Candida glabrata* CBS 138]	33572	320	6	6	24
gi|50284865	hypothetical protein [*Candida glabrata* CBS 138]	62304	292	7	7	19
gi|50284869	hypothetical protein [*Candida glabrata* CBS 138]	30312	277	6	6	21
gi|50295070	hypothetical protein [*Candida glabrata* CBS 138]	61157	269	6	5	12
gi|50295024	hypothetical protein [*Candida glabrata* CBS 138]	53878	220	5	5	13
gi|8927040	elongation factor 2 [*Candida glabrata*]	90394	196	3	3	4
gi|50284959	hypothetical protein [*Candida glabrata* CBS 138]	93783	196	3	3	3
gi|50293465	hypothetical protein [*Candida glabrata* CBS 138]	19705	187	5	3	13
gi|50284809	60S ribosomal protein L30 [*Candida glabrata* CBS 138]	11362	186	4	4	40
gi|50294171	hypothetical protein [*Candida glabrata* CBS 138]	30205	179	5	3	19
gi|18073449	GAS-1 homologue [*Candida glabrata*]	60675	176	5	4	9
gi|50287895	hypothetical protein [*Candida glabrata* CBS 138]	60701	176	5	4	9
gi|302309608	hypothetical protein [*Candida glabrata* CBS 138]	66558	168	5	5	12

gi|9650673	actin [*Candida glabrata*]	36600	158	7	6	21
gi|50288417	hypothetical protein [*Candida glabrata* CBS 138]	12784	130	2	2	20
gi|50286375	hypothetical protein [*Candida glabrata* CBS 138]	42980	121	3	3	7
gi|50292739	acetate--CoA ligase [*Candida glabrata* CBS 138]	79670	94	3	3	4
gi|50291857	40S ribosomal protein S20 [*Candida glabrata* CBS 138]	13547	90	2	2	19
gi|50286153	hypothetical protein [*Candida glabrata* CBS 138]	30297	88	2	2	9
gi|50287735	hypothetical protein [*Candida glabrata* CBS 138]	20710	87	2	2	13
gi|50286193	hypothetical protein [*Candida glabrata* CBS 138]	72967	85	3	2	4
gi|50286005	hypothetical protein [*Candida glabrata* CBS 138]	80047	82	3	2	3
gi|50290529	hypothetical protein [*Candida glabrata* CBS 138]	19235	69	3	2	13
gi|20258069	phospholipase B [*Candida glabrata*]	72217	69	2	2	2
gi|50291585	hypothetical protein [*Candida glabrata* CBS 138]	72055	69	2	2	2
gi|50294762	60S ribosomal protein L14 [*Candida glabrata* CBS 138]	15238	69	2	2	21
gi|50286667	hypothetical protein [*Candida glabrata* CBS 138]	39253	66	2	2	6
gi|50284831	hypothetical protein [*Candida glabrata* CBS 138]	63675	65	2	2	6
gi|50284867	hypothetical protein [*Candida glabrata* CBS 138]	61973	65	2	2	6
gi|50289975	hypothetical protein [*Candida glabrata* CBS 138]	20698	62	2	2	11
gi|50291341	hypothetical protein [*Candida glabrata* CBS 138]	20587	62	2	2	11
gi|50287337	60S ribosomal protein L12 [*Candida glabrata* CBS 138]	17799	62	3	3	17
gi|50288355	hypothetical protein [*Candida glabrata* CBS 138]	28757	58	2	2	10
**vesicles sonication 2**						

gi|50288681	hypothetical protein [*Candida glabrata* CBS 138]	35985	621	22	13	46
gi|50289857	hypothetical protein [*Candida glabrata* CBS 138]	46767	598	12	9	25
gi|50286669	hypothetical protein [*Candida glabrata* CBS 138]	59098	548	17	10	19
gi|50292035	hypothetical protein [*Candida glabrata* CBS 138]	136648	536	18	13	13
gi|50287897	hypothetical protein [*Candida glabrata* CBS 138]	39767	508	13	9	32
gi|25992752	pyruvate decarboxylase [*Candida glabrata*]	62011	443	13	12	26
gi|50294560	hypothetical protein [*Candida glabrata* CBS 138]	62152	443	13	12	26
gi|50290013	hypothetical protein [*Candida glabrata* CBS 138]	44930	416	12	10	29
gi|50289685	hypothetical protein [*Candida glabrata* CBS 138]	51468	387	9	8	18
gi|50285407	hypothetical protein [*Candida glabrata* CBS 138]	29899	296	8	8	40
gi|50291073	hypothetical protein [*Candida glabrata* CBS 138]	37779	272	6	5	15
gi|50295024	hypothetical protein [*Candida glabrata* CBS 138]	53878	267	5	5	13
gi|18073449	GAS-1 homologue [*Candida glabrata*]	60675	234	6	5	11
gi|50295070	hypothetical protein [*Candida glabrata* CBS 138]	61157	228	7	4	9
gi|50294908	hypothetical protein [*Candida glabrata* CBS 138]	54975	223	7	7	17
gi|50293403	hypothetical protein [*Candida glabrata* CBS 138]	44704	207	6	6	19
gi|50290317	hypothetical protein [*Candida glabrata* CBS 138]	37920	192	3	3	9
gi|20258069	phospholipase B [*Candida glabrata*]	72217	187	4	4	6
gi|50292597	hypothetical protein [*Candida glabrata* CBS 138]	20962	139	4	4	15
gi|452816300	acid trehalase [*Candida glabrata*]	136605	134	5	5	4
gi|50294025	hypothetical protein [*Candida glabrata* CBS 138]	43265	131	2	2	5

gi|50287737	hypothetical protein [*Candida glabrata* CBS 138]	21335	126	3	3	20
gi|50289459	hypothetical protein [*Candida glabrata* CBS 138]	26968	124	4	4	18
gi|50284733	hypothetical protein [*Candida glabrata* CBS 138]	98941	111	3	3	3
gi|50287951	60S acidic ribosomal protein P0 [*Candida glabrata* CBS 138]	33572	104	3	3	11
gi|50294171	hypothetical protein [*Candida glabrata* CBS 138]	30205	87	3	2	11
gi|50287735	hypothetical protein [*Candida glabrata* CBS 138]	20710	82	3	3	21
gi|50288349	hypothetical protein [*Candida glabrata* CBS 138]	32424	71	3	3	8
**vesicles sonication 3**						
gi|50286669	hypothetical protein [*Candida glabrata* CBS 138]	59098	369	9	7	17
gi|50288681	hypothetical protein [*Candida glabrata* CBS 138]	35985	310	10	9	35
gi|50294908	hypothetical protein [*Candida glabrata* CBS 138]	54975	295	10	10	24
gi|50292035	hypothetical protein [*Candida glabrata* CBS 138]	136648	291	8	8	7
gi|452816300	acid trehalase [*Candida glabrata*]	136605	291	8	8	7
gi|50288687	hypothetical protein [*Candida glabrata* CBS 138]	49013	259	8	8	21
gi|50291073	hypothetical protein [*Candida glabrata* CBS 138]	37779	258	7	6	23
gi|50293403	hypothetical protein [*Candida glabrata* CBS 138]	44704	238	7	7	19
gi|50290317	hypothetical protein [*Candida glabrata* CBS 138]	37920	216	5	4	13
gi|50289685	hypothetical protein [*Candida glabrata* CBS 138]	51468	208	6	6	13
gi|25992752	pyruvate decarboxylase [*Candida glabrata*]	62011	152	4	4	8
gi|50294560	hypothetical protein [*Candida glabrata* CBS 138]	62152	152	4	4	8
gi|50289857	hypothetical protein [*Candida glabrata* CBS 138]	46767	137	3	3	8

gi|50294025	hypothetical protein [*Candida glabrata* CBS 138]	43265	103	2	2	5
gi|50295070	hypothetical protein [*Candida glabrata* CBS 138]	61157	88	3	3	6
gi|50287897	hypothetical protein [*Candida glabrata* CBS 138]	39767	84	2	2	6
gi|20258069	phospholipase B [*Candida glabrata*]	72217	65	2	2	3
gi|50291585	hypothetical protein [*Candida glabrata* CBS 138]	72055	65	2	2	3
gi|50286919	hypothetical protein [*Candida glabrata* CBS 138]	44251	57	2	2	4
gi|50292739	acetate-CoA ligase [*Candida glabrata* CBS 138]	79670	55	1	1	1
**fraction enriched with membrane proteins 1**						
gi|50288681	hypothetical protein [*Candida glabrata* CBS 138]	35985	599	14	9	31
gi|50292035	hypothetical protein [*Candida glabrata* CBS 138]	136648	516	18	11	11
gi|50286669	hypothetical protein [*Candida glabrata* CBS 138]	59098	497	14	9	21
gi|50289685	hypothetical protein [*Candida glabrata* CBS 138]	51468	437	13	11	21
gi|50289857	hypothetical protein [*Candida glabrata* CBS 138]	46767	420	9	8	24
gi|50294908	hypothetical protein [*Candida glabrata* CBS 138]	54975	398	9	9	20
gi|50284959	hypothetical protein [*Candida glabrata* CBS 138]	93783	356	8	8	11
gi|50290317	hypothetical protein [*Candida glabrata* CBS 138]	37920	330	7	7	24
gi|50284865	hypothetical protein [*Candida glabrata* CBS 138]	62304	313	6	5	10
gi|50284733	hypothetical protein [*Candida glabrata* CBS 138]	98941	302	6	6	7
gi|50294560	hypothetical protein [*Candida glabrata* CBS 138]	62152	297	9	9	21
gi|50293403	hypothetical protein [*Candida glabrata* CBS 138]	44704	295	7	7	27
gi|50284867	hypothetical protein [*Candida glabrata* CBS 138]	61973	287	5	5	9

gi|50287897	hypothetical protein [*Candida glabrata* CBS 138]	39767	255	6	4	17
gi|50295024	hypothetical protein [*Candida glabrata* CBS 138]	53878	205	4	4	12
gi|50289283	hypothetical protein [*Candida glabrata* CBS 138]	42203	194	4	4	13
gi|50291073	hypothetical protein [*Candida glabrata* CBS 138]	37779	183	5	3	10
gi|50292739	acetate--CoA ligase [*Candida glabrata* CBS 138]	79670	164	3	3	5
gi|50294171	hypothetical protein [*Candida glabrata* CBS 138]	30205	152	4	3	17
gi|50288687	hypothetical protein [*Candida glabrata* CBS 138]	49013	137	5	5	15
gi|50284751	hypothetical protein [*Candida glabrata* CBS 138]	62484	133	2	2	3
gi|50293465	hypothetical protein [*Candida glabrata* CBS 138]	19705	90	1	1	7
gi|50286375	hypothetical protein [*Candida glabrata* CBS 138]	42980	89	4	3	11
gi|302309608	hypothetical protein [*Candida glabrata* CBS 138]	66558	85	3	3	5
gi|50287007	hypothetical protein [*Candida glabrata* CBS 138]	66848	78	2	2	3
gi|50292893	hypothetical protein [*Candida glabrata* CBS 138]	39503	73	2	2	4
gi|50287735	hypothetical protein [*Candida glabrata* CBS 138]	20710	65	2	2	16
gi|50286871	hypothetical protein [*Candida glabrata* CBS 138]	73467	65	1	1	2
gi|50285407	hypothetical protein [*Candida glabrata* CBS 138]	29899	61	2	2	9
gi|9650673	actin [*Candida glabrata*]	36600	57	2	2	7
**fraction enriched with membrane proteins 2**						
gi|50284733	hypothetical protein [*Candida glabrata* CBS 138]	98941	400	7	7	8
gi|50284865	hypothetical protein [*Candida glabrata* CBS 138]	62304	374	10	7	18
gi|50288681	hypothetical protein [*Candida glabrata* CBS 138]	35985	351	8	6	26

gi|25992752	pyruvate decarboxylase [*Candida glabrata*]	62011	206	5	5	11
gi|50289857	hypothetical protein [*Candida glabrata* CBS 138]	46767	205	4	3	9
gi|50294908	hypothetical protein [*Candida glabrata* CBS 138]	54975	189	4	4	10
gi|50284867	hypothetical protein [*Candida glabrata* CBS 138]	61973	184	5	4	8
gi|302309608	hypothetical protein [*Candida glabrata* CBS 138]	66558	153	5	5	10
gi|50286669	hypothetical protein [*Candida glabrata* CBS 138]	59098	149	5	3	6
gi|50292035	hypothetical protein [*Candida glabrata* CBS 138]	136648	140	4	4	4
gi|50286871	hypothetical protein [*Candida glabrata* CBS 138]	73467	138	3	3	5
gi|50293403	hypothetical protein [*Candida glabrata* CBS 138]	44704	110	2	2	5
gi|50292213	hypothetical protein [*Candida glabrata* CBS 138]	77437	95	3	3	4
gi|50289685	hypothetical protein [*Candida glabrata* CBS 138]	51468	81	2	2	5
gi|50287107	hypothetical protein [*Candida glabrata* CBS 138]	61626	76	2	2	3
gi|50284869	hypothetical protein [*Candida glabrata* CBS 138]	30312	75	2	2	5
gi|50284959	hypothetical protein [*Candida glabrata* CBS 138]	93783	75	3	3	3
gi|50286375	hypothetical protein [*Candida glabrata* CBS 138]	42980	72	4	3	11
gi|50295024	hypothetical protein [*Candida glabrata* CBS 138]	53878	65	1	1	2
gi|50288349	hypothetical protein [*Candida glabrata* CBS 138]	32424	63	1	1	3
gi|50292597	hypothetical protein [*Candida glabrata* CBS 138]	20962	58	2	2	7
gi|50288355	hypothetical protein [*Candida glabrata* CBS 138]	28757	55	2	1	7
**fraction enriched with membrane proteins 3**						
gi|50288681	hypothetical protein [*Candida glabrata* CBS 138]	35985	365	7	6	19

gi|50284733	hypothetical protein [*Candida glabrata* CBS 138]	98941	302	6	6	8
gi|50286669	hypothetical protein [*Candida glabrata* CBS 138]	59098	248	7	5	11
gi|50284865	hypothetical protein [*Candida glabrata* CBS 138]	62304	228	6	5	13
gi|50284867	hypothetical protein [*Candida glabrata* CBS 138]	61973	214	5	5	12
gi|302309608	hypothetical protein [*Candida glabrata* CBS 138]	66558	207	5	5	10
gi|50289857	hypothetical protein [*Candida glabrata* CBS 138]	46767	184	3	3	11
gi|50294908	hypothetical protein [*Candida glabrata* CBS 138]	54975	166	3	3	8
gi|50294560	hypothetical protein [*Candida glabrata* CBS 138]	62152	161	5	5	9
gi|50295024	hypothetical protein [*Candida glabrata* CBS 138]	53878	156	2	2	5
gi|50286871	hypothetical protein [*Candida glabrata* CBS 138]	73467	151	3	3	5
gi|50287737	hypothetical protein [*Candida glabrata* CBS 138]	21335	148	3	3	23
gi|50293403	hypothetical protein [*Candida glabrata* CBS 138]	44704	134	2	2	5
gi|50284959	hypothetical protein [*Candida glabrata* CBS 138]	93783	133	4	4	4
gi|50290317	hypothetical protein [*Candida glabrata* CBS 138]	37920	109	2	2	6
gi|50287007	hypothetical protein [*Candida glabrata* CBS 138]	66848	97	2	1	2
gi|50292035	hypothetical protein [*Candida glabrata* CBS 138]	136648	96	4	3	2
gi|50284875	hypothetical protein [*Candida glabrata* CBS 138]	62161	89	1	1	2
gi|50284869	hypothetical protein [*Candida glabrata* CBS 138]	30312	80	2	2	5
gi|50290935	hypothetical protein [*Candida glabrata* CBS 138]	29748	78	2	1	6
gi|50287107	hypothetical protein [*Candida glabrata* CBS 138]	61626	71	2	2	3
gi|50286005	hypothetical protein [*Candida glabrata* CBS 138]	80047	69	3	2	4

gi|50290059	hypothetical protein [*Candida glabrata* CBS 138]	171561	61	2	2	1
gi|50293465	hypothetical protein [*Candida glabrata* CBS 138]	19705	60	1	1	7
gi|50290013	hypothetical protein [*Candida glabrata* CBS 138]	44930	56	2	2	4
gi|50292893	hypothetical protein [*Candida glabrata* CBS 138]	39503	56	2	2	4
**fraction enriched with membrane proteins 4**						
gi|50288681	hypothetical protein [*Candida glabrata* CBS 138]	35985	508	14	11	45
gi|50293403	hypothetical protein [*Candida glabrata* CBS 138]	44704	483	15	14	43
gi|50289857	hypothetical protein [*Candida glabrata* CBS 138]	46767	430	9	8	23
gi|50286669	hypothetical protein [*Candida glabrata* CBS 138]	59098	363	11	7	12
gi|50292035	hypothetical protein [*Candida glabrata* CBS 138]	136648	357	11	11	9
gi|50288687	hypothetical protein [*Candida glabrata* CBS 138]	49013	295	8	8	20
gi|50289685	hypothetical protein [*Candida glabrata* CBS 138]	51468	282	9	9	18
gi|50289283	hypothetical protein [*Candida glabrata* CBS 138]	42203	214	4	4	12
gi|50284869	hypothetical protein [*Candida glabrata* CBS 138]	30312	191	4	4	16
gi|50290013	hypothetical protein [*Candida glabrata* CBS 138]	44930	189	5	5	14
gi|50287897	hypothetical protein [*Candida glabrata* CBS 138]	39767	179	4	4	15
gi|50295024	hypothetical protein [*Candida glabrata* CBS 138]	53878	166	4	4	10
gi|50294908	hypothetical protein [*Candida glabrata* CBS 138]	54975	164	5	5	11
gi|50287951	60S acidic ribosomal protein P0 [*Candida glabrata* CBS 138]	33572	156	5	4	15
gi|50290317	hypothetical protein [*Candida glabrata* CBS 138]	37920	154	3	3	10
gi|50284809	60S ribosomal protein L30 [*Candida glabrata* CBS 138]	11362	130	4	4	44

gi|50289459	hypothetical protein [*Candida glabrata* CBS 138]	26968	130	4	4	17
gi|50286153	hypothetical protein [*Candida glabrata* CBS 138]	30297	121	3	3	13
gi|25992752	pyruvate decarboxylase [*Candida glabrata*]	62011	116	3	3	6
gi|50294560	hypothetical protein [*Candida glabrata* CBS 138]	62152	116	3	3	6
gi|50292893	hypothetical protein [*Candida glabrata* CBS 138]	39503	104	3	3	9
gi|50287735	hypothetical protein [*Candida glabrata* CBS 138]	20710	84	2	2	13
gi|50294025	hypothetical protein [*Candida glabrata* CBS 138]	43265	80	2	2	5
gi|50285407	hypothetical protein [*Candida glabrata* CBS 138]	29899	69	3	3	11
gi|50287073	hypothetical protein [*Candida glabrata* CBS 138]	27574	68	2	2	8
gi|50286375	hypothetical protein [*Candida glabrata* CBS 138]	42980	66	1	1	2
gi|50292739	acetate--CoA ligase [*Candida glabrata* CBS 138]	79670	61	1	1	1
gi|50284959	hypothetical protein [*Candida glabrata* CBS 138]	93783	57	2	2	2
