# Supplementary material for: Characteristics of Extracellular Vesicles Released by the Pathogenic Yeast-Like Fungi Candida glabrata, Candida parapsilosis and Candida tropicalis
Source: Cells. 2020 Jul 18;9(7):1722. doi: 10.3390/cells9071722 (PMC7408413; doi:10.3390/cells9071722)
Supplement: Supplementary file 1 [file cells-09-01722-s001.zip › Supplementary table 2.pdf]

Supplementary Table 2. Mass spectrometry identification of *C. parapsilosis* proteins in EVs after vesicle surface shaving with trypsin, vesicle sonication, or the preparation of fractions enriched with membrane proteins.

The resulting peptides were analyzed using the Dionex Ultimate 3000 UHPLC system coupled to an HCTUltra ETDII mass spectrometer and the obtained lists of peaks were searched against the NCBI protein database using an in-house Mascot server.

Accession number	Protein	Molecular mass [Da]	Score	Matches	Sequences	Sequence coverage [%]
**surface shaving with trypsin 1**						
gi|354544096	hypothetical protein CPAR2_108560 [*Candida parapsilosis*]	44734	434	10	7	19
gi|354547623	hypothetical protein CPAR2_401600 [*Candida parapsilosis*]	34120	398	10	8	22
gi|354546478	hypothetical protein CPAR2_208530 [*Candida parapsilosis*]	35667	327	7	6	26
gi|354548190	hypothetical protein CPAR2_407280 [*Candida parapsilosis*]	30559	270	6	5	30
gi|354548203	hypothetical protein CPAR2_407410 [*Candida parapsilosis*]	39522	258	6	4	12
gi|354543842	hypothetical protein CPAR2_106000 [*Candida parapsilosis*]	49102	256	7	6	21
gi|354543893	hypothetical protein CPAR2_106500 [*Candida parapsilosis*]	56211	245	6	5	12
gi|354544089	hypothetical protein CPAR2_108490 [*Candida parapsilosis*]	38798	199	6	5	17
gi|354545372	hypothetical protein CPAR2_806490 [*Candida parapsilosis*]	41477	190	3	3	7
gi|354544910	hypothetical protein CPAR2_801850 [*Candida parapsilosis*]	41980	182	4	2	9
gi|354545518	hypothetical protein CPAR2_807950 [*Candida parapsilosis*]	45323	163	4	4	11
gi|354544206	hypothetical protein CPAR2_109660 [*Candida parapsilosis*]	60797	137	2	2	4
gi|354545390	hypothetical protein CPAR2_806670 [*Candida parapsilosis*]	53439	129	6	2	5

gi|354544345	hypothetical protein CPAR2_300570 [*Candida parapsilosis*]	31701	114	2	2	7
gi|354543504	hypothetical protein CPAR2_102610 [*Candida parapsilosis*]	64176	114	2	2	6
gi|354548052	hypothetical protein CPAR2_405910 [*Candida parapsilosis*]	32870	111	3	3	10
gi|354547257	hypothetical protein CPAR2_502160 [*Candida parapsilosis*]	49909	101	2	2	5
gi|354547091	hypothetical protein CPAR2_500500 [*Candida parapsilosis*]	36553	89	1	1	4
gi|354543976	hypothetical protein CPAR2_107330 [*Candida parapsilosis*]	37592	87	2	2	10
gi|354544883	hypothetical protein CPAR2_801600 [*Candida parapsilosis*]	99191	65	2	2	2
gi|354545228	hypothetical protein CPAR2_805040 [*Candida parapsilosis*]	23446	56	1	1	4
gi|354547939	hypothetical protein CPAR2_404780 [*Candida parapsilosis*]	114085	55	1	1	1
gi|354547941	hypothetical protein CPAR2_404800 [*Candida parapsilosis*]	144019	55	1	1	1
**surface shaving with trypsin 2**						
gi|354544096	hypothetical protein CPAR2_108560 [*Candida parapsilosis*]	44734	541	10	9	22
gi|354547623	hypothetical protein CPAR2_401600 [*Candida parapsilosis*]	34120	407	10	8	31
gi|354543893	hypothetical protein CPAR2_106500 [*Candida parapsilosis*]	56211	358	8	7	16
gi|354546478	hypothetical protein CPAR2_208530 [*Candida parapsilosis*]	35667	351	7	5	21
gi|354543842	hypothetical protein CPAR2_106000 [*Candida parapsilosis*]	49102	329	7	6	19
gi|354548190	hypothetical protein CPAR2_407280 [*Candida parapsilosis*]	30559	279	8	5	24
gi|354548203	hypothetical protein CPAR2_407410 [*Candida parapsilosis*]	39522	197	8	4	9
gi|354543976	hypothetical protein CPAR2_107330 [*Candida parapsilosis*]	37592	179	3	3	12
gi|354545372	hypothetical protein CPAR2_806490 [*Candida parapsilosis*]	41477	179	3	3	7
gi|354548052	hypothetical protein CPAR2_405910 [*Candida parapsilosis*]	32870	168	4	4	11

gi|354547356	hypothetical protein CPAR2_503160 [*Candida parapsilosis*]	51464	165	4	4	10
gi|354544206	hypothetical protein CPAR2_109660 [*Candida parapsilosis*]	60797	159	3	3	7
gi|354544345	hypothetical protein CPAR2_300570 [*Candida parapsilosis*]	31701	151	3	3	9
gi|354543610	hypothetical protein CPAR2_103690 [*Candida parapsilosis*]	60638	146	3	3	7
gi|354548638	hypothetical protein CPAR2_703880 [*Candida parapsilosis*]	92640	132	4	4	7
gi|354545390	hypothetical protein CPAR2_806670 [*Candida parapsilosis*]	53439	131	5	2	5
gi|354547257	hypothetical protein CPAR2_502160 [*Candida parapsilosis*]	49909	126	2	2	6
gi|354545228	hypothetical protein CPAR2_805040 [*Candida parapsilosis*]	23446	96	3	2	7
gi|354544910	hypothetical protein CPAR2_801850 [*Candida parapsilosis*]	41980	89	7	1	3
gi|354547091	hypothetical protein CPAR2_500500 [*Candida parapsilosis*]	36553	83	1	1	4
gi|354548067	hypothetical protein CPAR2_406060 [*Candida parapsilosis*]	84922	78	2	2	4
gi|354544883	hypothetical protein CPAR2_801600 [*Candida parapsilosis*]	99191	75	2	2	2
gi|354543315	hypothetical protein CPAR2_100710 [*Candida parapsilosis*]	47017	74	1	1	2
gi|354547549	hypothetical protein CPAR2_400860 [*Candida parapsilosis*]	52009	69	1	1	2
gi|354544089	hypothetical protein CPAR2_108490 [*Candida parapsilosis*]	38798	65	2	2	5
gi|354546504	hypothetical protein CPAR2_208800 [*Candida parapsilosis*]	35461	63	1	1	3
gi|354545518	hypothetical protein CPAR2_807950 [*Candida parapsilosis*]	45323	61	1	1	2
gi|354543994	hypothetical protein CPAR2_107510 [*Candida parapsilosis*]	64136	58	1	1	2
gi|354548402	hypothetical protein CPAR2_701420 [*Candida parapsilosis*]	125716	57	1	1	1
**surface shaving with trypsin 3**						
gi|354547623	hypothetical protein CPAR2_401600 [*Candida parapsilosis*]	34120	347	7	6	17

gi|354546478	hypothetical protein CPAR2_208530 [*Candida parapsilosis*]	35667	334	5	5	22
gi|354548203	hypothetical protein CPAR2_407410 [*Candida parapsilosis*]	39522	243	5	4	12
gi|354543842	hypothetical protein CPAR2_106000 [*Candida parapsilosis*]	49102	232	5	5	12
gi|354543893	hypothetical protein CPAR2_106500 [*Candida parapsilosis*]	56211	231	4	4	10
gi|354545372	hypothetical protein CPAR2_806490 [*Candida parapsilosis*]	41477	177	3	3	7
gi|354548052	hypothetical protein CPAR2_405910 [*Candida parapsilosis*]	32870	135	4	4	12
gi|354548190	hypothetical protein CPAR2_407280 [*Candida parapsilosis*]	30559	132	3	3	8
gi|354544089	hypothetical protein CPAR2_108490 [*Candida parapsilosis*]	38798	126	5	4	13
gi|354545390	hypothetical protein CPAR2_806670 [*Candida parapsilosis*]	53439	126	3	2	5
gi|354544910	hypothetical protein CPAR2_801850 [*Candida parapsilosis*]	41980	122	4	2	9
gi|354544345	hypothetical protein CPAR2_300570 [*Candida parapsilosis*]	31701	107	3	3	12
gi|354543610	hypothetical protein CPAR2_103690 [*Candida parapsilosis*]	60638	105	3	2	6
gi|354548638	hypothetical protein CPAR2_703880 [*Candida parapsilosis*]	92640	85	2	2	2
gi|354544096	hypothetical protein CPAR2_108560 [*Candida parapsilosis*]	44734	83	2	2	6
gi|354544206	hypothetical protein CPAR2_109660 [*Candida parapsilosis*]	60797	64	1	1	1
gi|354545518	hypothetical protein CPAR2_807950 [*Candida parapsilosis*]	45323	60	1	1	2
gi|354543994	hypothetical protein CPAR2_107510 [*Candida parapsilosis*]	64136	59	1	1	1
gi|354545228	hypothetical protein CPAR2_805040 [*Candida parapsilosis*]	23446	56	1	1	4
**surface shaving with trypsin 4**						
gi|354547623	hypothetical protein CPAR2_401600 [*Candida parapsilosis*]	34120	373	10	7	22
gi|354543893	hypothetical protein CPAR2_106500 [*Candida parapsilosis*]	56211	291	7	6	15

gi|354543842	hypothetical protein CPAR2_106000 [*Candida parapsilosis*]	49102	284	8	6	16
gi|354548203	hypothetical protein CPAR2_407410 [*Candida parapsilosis*]	39522	209	5	4	12
gi|354548052	hypothetical protein CPAR2_405910 [*Candida parapsilosis*]	32870	111	2	2	6
gi|354546478	hypothetical protein CPAR2_208530 [*Candida parapsilosis*]	35667	110	3	3	12
gi|354548511	hypothetical protein CPAR2_702600 [*Candida parapsilosis*]	28538	110	5	3	14
gi|354548638	hypothetical protein CPAR2_703880 [*Candida parapsilosis*]	92640	106	3	3	4
gi|354544883	hypothetical protein CPAR2_801600 [*Candida parapsilosis*]	99191	104	3	3	3
gi|354544345	hypothetical protein CPAR2_300570 [*Candida parapsilosis*]	31701	84	2	2	7
gi|354548190	hypothetical protein CPAR2_407280 [*Candida parapsilosis*]	30559	81	2	2	10
gi|354544804	hypothetical protein CPAR2_800810 [*Candida parapsilosis*]	52103	68	4	4	10
gi|354545590	hypothetical protein CPAR2_808670 [*Candida parapsilosis*]	36264	63	2	2	5
gi|354544910	hypothetical protein CPAR2_801850 [*Candida parapsilosis*]	41980	54	1	1	3
gi|354546348	hypothetical protein CPAR2_207210 [*Candida parapsilosis*]	46995	53	1	1	3
**vesicles sonication 1**						
gi|354544127	hypothetical protein CPAR2_108890 [*Candida parapsilosis*]	118910	583	16	13	14
gi|354548203	hypothetical protein CPAR2_407410 [*Candida parapsilosis*]	39522	459	8	8	26
gi|354547623	hypothetical protein CPAR2_401600 [*Candida parapsilosis*]	34120	419	11	8	25
gi|354548190	hypothetical protein CPAR2_407280 [*Candida parapsilosis*]	30559	350	13	7	30
gi|354547091	hypothetical protein CPAR2_500500 [*Candida parapsilosis*]	36553	253	6	3	11
gi|354543976	hypothetical protein CPAR2_107330 [*Candida parapsilosis*]	37592	232	4	4	16
gi|354547178	hypothetical protein CPAR2_501380 [*Candida parapsilosis*]	49439	230	7	7	18

gi|354547255	hypothetical protein CPAR2_502140 [*Candida parapsilosis*]	61228	221	5	3	7
gi|354545228	hypothetical protein CPAR2_805040 [*Candida parapsilosis*]	23446	220	6	3	13
gi|354543255	hypothetical protein CPAR2_100110 [*Candida parapsilosis*]	50537	214	7	5	17
gi|354544501	hypothetical protein CPAR2_302140 [*Candida parapsilosis*]	60168	214	5	5	10
gi|354544096	hypothetical protein CPAR2_108560 [*Candida parapsilosis*]	44734	164	4	4	8
gi|354544206	hypothetical protein CPAR2_109660 [*Candida parapsilosis*]	60797	162	4	3	7
gi|354545518	hypothetical protein CPAR2_807950 [*Candida parapsilosis*]	45323	151	3	3	8
gi|354543893	hypothetical protein CPAR2_106500 [*Candida parapsilosis*]	56211	135	3	3	5
gi|354543404	hypothetical protein CPAR2_101610 [*Candida parapsilosis*]	57858	134	5	4	9
gi|354545390	hypothetical protein CPAR2_806670 [*Candida parapsilosis*]	53439	131	5	2	5
gi|354544910	hypothetical protein CPAR2_801850 [*Candida parapsilosis*]	41980	115	2	1	3
gi|354544403	hypothetical protein CPAR2_301150 [*Candida parapsilosis*]	32011	109	4	4	24
gi|354544345	hypothetical protein CPAR2_300570 [*Candida parapsilosis*]	31701	92	3	3	12
gi|354545372	hypothetical protein CPAR2_806490 [*Candida parapsilosis*]	41477	88	2	2	4
gi|21953342	lipase 2 [*Candida parapsilosis*]	51104	80	4	4	11
gi|354547299	hypothetical protein CPAR2_502580 [*Candida parapsilosis*]	43513	80	3	3	8
gi|354548325	hypothetical protein CPAR2_700650 [*Candida parapsilosis*]	34248	75	2	2	6
gi|354547664	hypothetical protein CPAR2_402000 [*Candida parapsilosis*]	29933	56	2	2	8
gi|354547939	hypothetical protein CPAR2_404780 [*Candida parapsilosis*]	114085	54	2	1	1
**vesicles sonication 2**						
gi|354544127	hypothetical protein CPAR2_108890 [*Candida parapsilosis*]	118910	583	13	12	12

gi|354547623	hypothetical protein CPAR2_401600 [*Candida parapsilosis*]	34120	451	10	9	26
gi|354548203	hypothetical protein CPAR2_407410 [*Candida parapsilosis*]	39522	327	6	5	17
gi|354547255	hypothetical protein CPAR2_502140 [*Candida parapsilosis*]	61228	296	7	4	10
gi|354548190	hypothetical protein CPAR2_407280 [*Candida parapsilosis*]	30559	279	7	4	22
gi|354547091	hypothetical protein CPAR2_500500 [*Candida parapsilosis*]	36553	265	6	3	11
gi|354547257	hypothetical protein CPAR2_502160 [*Candida parapsilosis*]	49909	241	7	6	21
gi|354544206	hypothetical protein CPAR2_109660 [*Candida parapsilosis*]	60797	204	5	5	11
gi|354543976	hypothetical protein CPAR2_107330 [*Candida parapsilosis*]	37592	192	4	3	12
gi|354545518	hypothetical protein CPAR2_807950 [*Candida parapsilosis*]	45323	182	3	3	8
gi|354543893	hypothetical protein CPAR2_106500 [*Candida parapsilosis*]	56211	177	7	5	11
gi|354545228	hypothetical protein CPAR2_805040 [*Candida parapsilosis*]	23446	166	4	3	13
gi|354545390	hypothetical protein CPAR2_806670 [*Candida parapsilosis*]	53439	150	7	2	5
gi|354548325	hypothetical protein CPAR2_700650 [*Candida parapsilosis*]	34248	142	3	3	11
gi|354543255	hypothetical protein CPAR2_100110 [*Candida parapsilosis*]	50537	105	3	3	10
gi|354544403	hypothetical protein CPAR2_301150 [*Candida parapsilosis*]	32011	90	4	4	13
gi|354544910	hypothetical protein CPAR2_801850 [*Candida parapsilosis*]	41980	87	5	1	3
gi|354543610	hypothetical protein CPAR2_103690 [*Candida parapsilosis*]	60638	59	2	2	4
**vesicles sonication 3**						
gi|354544804	hypothetical protein CPAR2_800810 [*Candida parapsilosis*]	52103	754	17	13	34
gi|354544127	hypothetical protein CPAR2_108890 [*Candida parapsilosis*]	118910	662	12	12	12
gi|354547623	hypothetical protein CPAR2_401600 [*Candida parapsilosis*]	34120	655	15	11	31

gi|354548190	hypothetical protein CPAR2_407280 [*Candida parapsilosis*]	30559	583	18	9	40
gi|354548203	hypothetical protein CPAR2_407410 [*Candida parapsilosis*]	39522	521	9	7	26
gi|354544096	hypothetical protein CPAR2_108560 [*Candida parapsilosis*]	44734	493	9	8	19
gi|354546478	hypothetical protein CPAR2_208530 [*Candida parapsilosis*]	35667	462	10	8	31
gi|354544206	hypothetical protein CPAR2_109660 [*Candida parapsilosis*]	60797	455	8	7	14
gi|354543842	hypothetical protein CPAR2_106000 [*Candida parapsilosis*]	49102	454	11	9	30
gi|354547255	hypothetical protein CPAR2_502140 [*Candida parapsilosis*]	61228	371	8	5	10
gi|354543255	hypothetical protein CPAR2_100110 [*Candida parapsilosis*]	50537	334	7	6	20
gi|354547257	hypothetical protein CPAR2_502160 [*Candida parapsilosis*]	49909	333	7	5	13
gi|354543976	hypothetical protein CPAR2_107330 [*Candida parapsilosis*]	37592	325	6	5	20
gi|354543893	hypothetical protein CPAR2_106500 [*Candida parapsilosis*]	56211	316	7	5	10
gi|354545518	hypothetical protein CPAR2_807950 [*Candida parapsilosis*]	45323	285	6	5	21
gi|354545372	hypothetical protein CPAR2_806490 [*Candida parapsilosis*]	41477	272	4	4	9
gi|354544501	hypothetical protein CPAR2_302140 [*Candida parapsilosis*]	60168	259	5	5	10
gi|354544089	hypothetical protein CPAR2_108490 [*Candida parapsilosis*]	38798	245	6	6	17
gi|354547091	hypothetical protein CPAR2_500500 [*Candida parapsilosis*]	36553	244	9	3	11
gi|354548638	hypothetical protein CPAR2_703880 [*Candida parapsilosis*]	92640	214	7	7	8
gi|354543504	hypothetical protein CPAR2_102610 [*Candida parapsilosis*]:	64176	213	5	4	7
gi|354545228	hypothetical protein CPAR2_805040 [*Candida parapsilosis*]	23446	201	5	3	13
gi|354546348	hypothetical protein CPAR2_207210 [*Candida parapsilosis*]	46995	192	4	4	12
gi|354548052	hypothetical protein CPAR2_405910 [*Candida parapsilosis*]	32870	188	4	4	13

gi|354548325	hypothetical protein CPAR2_700650 [*Candida parapsilosis*]	34248	173	3	3	12
gi|354545390	hypothetical protein CPAR2_806670 [*Candida parapsilosis*]	53439	167	7	2	5
gi|354544345	hypothetical protein CPAR2_300570 [*Candida parapsilosis*]	31701	166	4	4	14
gi|354544418	hypothetical protein CPAR2_301300 [*Candida parapsilosis*]:	44831	160	4	4	10
gi|354547664	hypothetical protein CPAR2_402000 [*Candida parapsilosis*]	29933	147	4	4	15
gi|354547665	hypothetical protein CPAR2_402010 [*Candida parapsilosis*]	28561	147	4	4	16
gi|21953342	lipase 2 [*Candida parapsilosis*]	51104	147	4	4	9
gi|354548185	hypothetical protein CPAR2_407230 [*Candida parapsilosis*]	30718	143	6	3	9
gi|354548441	hypothetical protein CPAR2_701890 [*Candida parapsilosis*]	41314	142	4	4	11
gi|354546504	hypothetical protein CPAR2_208800 [*Candida parapsilosis*]	35461	142	2	2	8
gi|354547299	hypothetical protein CPAR2_502580 [*Candida parapsilosis*]	43513	141	3	3	7
gi|380005457	carbonyl reductase CPCR1 [*Candida parapsilosis*]	37536	141	3	3	9
gi|354545695	hypothetical protein CPAR2_200660 [*Candida parapsilosis*]	52061	140	3	3	6
gi|354547406	hypothetical protein CPAR2_503650 [*Candida parapsilosis*]	47288	136	2	2	6
gi|354544403	hypothetical protein CPAR2_301150 [*Candida parapsilosis*]	32011	129	4	4	19
gi|354543994	hypothetical protein CPAR2_107510 [*Candida parapsilosis*]	64136	125	4	4	6
gi|354544910	hypothetical protein CPAR2_801850 [*Candida parapsilosis*]	41980	119	8	1	3
gi|354543404	hypothetical protein CPAR2_101610 [*Candida parapsilosis*]	57858	109	3	3	7
gi|354545615	hypothetical protein CPAR2_808920 [*Candida parapsilosis*]	94691	103	2	2	2
gi|354543172	hypothetical protein CPAR2_603090 [*Candida parapsilosis*]	45622	93	3	3	10
gi|354545285	hypothetical protein CPAR2_805610 [*Candida parapsilosis*]	29776	89	2	2	8

gi|354547356	hypothetical protein CPAR2_503160 [*Candida parapsilosis*]	51464	82	2	2	6
gi|354547058	hypothetical protein CPAR2_500170 [*Candida parapsilosis*]	68788	80	2	2	2
gi|354543610	hypothetical protein CPAR2_103690 [*Candida parapsilosis*]	60638	79	2	2	4
gi|354543681	hypothetical protein CPAR2_104380 [*Candida parapsilosis*]	31394	76	3	3	13
gi|354548067	hypothetical protein CPAR2_406060 [*Candida parapsilosis*]	84922	70	3	3	5
gi|354547340	hypothetical protein CPAR2_502990 [*Candida parapsilosis*]	27451	65	2	2	8
gi|354547940	hypothetical protein CPAR2_404790 [*Candida parapsilosis*]	250245	58	1	1	0
gi|354547821	hypothetical protein CPAR2_403590 [*Candida parapsilosis*]	56088	55	2	2	3
**fraction enriched with membrane proteins 1**						
gi|354547623	hypothetical protein CPAR2_401600 [*Candida parapsilosis*]	34120	207	5	4	14
gi|354548203	hypothetical protein CPAR2_407410 [*Candida parapsilosis*]	39522	198	4	3	11
gi|354547091	hypothetical protein CPAR2_500500 [*Candida parapsilosis*]	36553	144	4	2	7
gi|354548190	hypothetical protein CPAR2_407280 [*Candida parapsilosis*]	30559	137	3	3	14
gi|354545390	hypothetical protein CPAR2_806670 [*Candida parapsilosis*]	53439	137	4	2	5
gi|354543976	hypothetical protein CPAR2_107330 [*Candida parapsilosis*]	37592	106	3	2	7
gi|354543893	hypothetical protein CPAR2_106500 [*Candida parapsilosis*]	56211	105	3	3	6
gi|354545228	hypothetical protein CPAR2_805040 [*Candida parapsilosis*]	23446	104	2	2	7
gi|354545372	hypothetical protein CPAR2_806490 [*Candida parapsilosis*]	41477	84	2	2	5
gi|354545518	hypothetical protein CPAR2_807950 [*Candida parapsilosis*]	45323	78	2	2	5
gi|354544096	hypothetical protein CPAR2_108560 [*Candida parapsilosis*]	44734	74	3	3	6
gi|354544910	hypothetical protein CPAR2_801850 [*Candida parapsilosis*]	41980	62	1	1	3

**fraction enriched with membrane proteins 2**						
gi|354548190	hypothetical protein CPAR2_407280 [*Candida parapsilosis*]	30559	424	13	8	32
gi|354548203	hypothetical protein CPAR2_407410 [*Candida parapsilosis*]	39522	259	5	5	17
gi|354547623	hypothetical protein CPAR2_401600 [*Candida parapsilosis*]	34120	235	6	5	16
gi|354545518	hypothetical protein CPAR2_807950 [*Candida parapsilosis*]	45323	217	4	4	8
gi|354544910	hypothetical protein CPAR2_801850 [*Candida parapsilosis*]	41980	216	8	3	11
gi|354543893	hypothetical protein CPAR2_106500 [*Candida parapsilosis*]	56211	211	5	4	9
gi|354543842	hypothetical protein CPAR2_106000 [*Candida parapsilosis*]	49102	160	4	4	11
gi|354545390	hypothetical protein CPAR2_806670 [*Candida parapsilosis*]	53439	131	4	2	5
gi|354544403	hypothetical protein CPAR2_301150 [*Candida parapsilosis*]	32011	129	4	3	15
gi|354544096	hypothetical protein CPAR2_108560 [*Candida parapsilosis*]	44734	118	5	3	9
gi|354545228	hypothetical protein CPAR2_805040 [*Candida parapsilosis*]	23446	100	2	2	7
gi|354546478	hypothetical protein CPAR2_208530 [*Candida parapsilosis*]	35667	96	2	2	8
gi|354546810	hypothetical protein CPAR2_211860 [*Candida parapsilosis*]	58004	88	2	2	2
gi|354548052	hypothetical protein CPAR2_405910 [*Candida parapsilosis*]	32870	68	3	3	9
gi|354546504	hypothetical protein CPAR2_208800 [*Candida parapsilosis*]	35461	62	1	1	3
gi|354544883	hypothetical protein CPAR2_801600 [*Candida parapsilosis*]	99191	59	1	1	1
gi|354548638	hypothetical protein CPAR2_703880 [*Candida parapsilosis*]	92640	56	1	1	1
**fraction enriched with membrane proteins 3**						
gi|354547623	hypothetical protein CPAR2_401600 [*Candida parapsilosis*]	34120	255	6	5	15
gi|354543976	hypothetical protein CPAR2_107330 [*Candida parapsilosis*]	37592	247	7	5	17

gi|354548190	hypothetical protein CPAR2_407280 [*Candida parapsilosis*]	30559	244	8	7	38
gi|354546478	hypothetical protein CPAR2_208530 [*Candida parapsilosis*]	35667	213	4	4	17
gi|354548203	hypothetical protein CPAR2_407410 [*Candida parapsilosis*]	39522	181	5	4	12
gi|354545390	hypothetical protein CPAR2_806670 [*Candida parapsilosis*]	53439	148	2	2	5
gi|354544910	hypothetical protein CPAR2_801850 [*Candida parapsilosis*]	41980	136	3	2	5
gi|354547091	hypothetical protein CPAR2_500500 [*Candida parapsilosis*]	36553	135	3	2	7
gi|354548052	hypothetical protein CPAR2_405910 [*Candida parapsilosis*]	32870	121	3	3	10
gi|354543893	hypothetical protein CPAR2_106500 [*Candida parapsilosis*]	56211	120	3	2	5
gi|354543610	hypothetical protein CPAR2_103690 [*Candida parapsilosis*]	60638	118	3	2	6
gi|354545518	hypothetical protein CPAR2_807950 [*Candida parapsilosis*]	45323	102	2	2	5
gi|354546810	hypothetical protein CPAR2_211860 [*Candida parapsilosis*]	58004	98	3	2	2
gi|354544089	hypothetical protein CPAR2_108490 [*Candida parapsilosis*]	38798	55	2	2	11
gi|354544403	hypothetical protein CPAR2_301150 [*Candida parapsilosis*]	32011	53	1	1	6
